# Untethered bistable origami crawler for confined applications

**DOI:** 10.1038/s44172-024-00294-1

**Published:** 2024-10-30

**Authors:** Catherine Jiayi Cai, Hui Huang, Hongliang Ren

**Affiliations:** 1 Department of Biomedical Engineering, National University of Singapore, Singapore, 117575 Singapore; 2 Singapore Institute of Manufacturing Technology, Agency for Science, Technology and Research (A*STAR), 5 Cleantech Loop, Singapore, 636732 Singapore; 3 Department of Electronic Engineering, Faculty of Engineering, The Chinese University of Hong Kong, Hong Kong, China; 4 Engineering Cluster, Singapore Institute of Technology, 10 Dover Drive, Singapore, 138683 Singapore; 5 Present Address: Department of Mechanical Engineering, National University of Singapore, Singapore, 117575 Singapore

**Keywords:** Biomedical engineering, Mechanical engineering

## Abstract

Magnetically actuated miniature origami crawlers are capable of robust locomotion in confined environments but are limited to passive functionalities. Here, we propose a bistable origami crawler that can shape-morph to access two separate regimes of folding degrees of freedom that are separated by an energy barrier. Using the modified bistable V-fold origami crease pattern as the fundamental unit of the crawler, we incorporated internal permanent magnets to enable untethered shape-morphing. By modulating the orientation of the external magnetic field, the crawler can reconfigure between an undeployed locomotion state and a deployed load-bearing state. In the undeployed state, the crawler can deform to enable out-of-plane crawling for robust bi-directional locomotion and navigation in confined environments based on friction anisotropy. In the deployed state, the crawler can execute microneedle insertion in confined environments. Through this work, we demonstrated the advantage of incorporating bistability into origami mechanisms to expand their capabilities in space-constraint applications.

## Abstract (web summary)

Catherine Jiayi Cai, Hui Huang and Hongliang Ren design a magnetically actuated bistable origami crawler that can transition between a locomotion and function mode, where each mode can be operated independently. The origami crawler is designed for use in confined spaces such as the gastrointestinal tract.

## Introduction

Untethered magnetically actuated millimeter-scale devices have huge potential and applications in confined environments like the gastrointestinal (GI) tract which are otherwise difficult to access for tethered and wired devices. In particular, magnetically actuated compliant origami devices are promising due to their ability to adapt their shape to achieve various tasks via folding and unfolding^1^, enabling the execution of sophisticated tasks in confined environments despite having limited on-board space. This can be attributed to their underlying folds and creases that impart degrees-of-freedoms (DoFs) in their underlying materials^2^. However, there is a dichotomy in the field whereby the folding DoFs of origami mechanisms give rise to unimodal deformations that are either utilized solely for locomotion or therapeutic functionalities, but never both.

For origami devices where unimodal deformation is utilized for functionalities, the geometry of origami mechanisms is frequently exploited for locomotion instead. For example, origami mechanisms based on the sarrus^3^ and kresing^4^ are designed to have a spherical or elliptical geometry that can be used for rigid-body rotational, translation, and rolling-based locomotion. The axial DoF conferred via deformation of the origami mechanism is then employed for active load-bearing biomedical interventions such as controlled delivery of liquid drugs^4,5^, and biopsy^6–8^. While such locomotion modes are sufficient in cavernous GI environments such as the stomach, they require large magnetic forces and magnetic field gradients to overcome resistive contact forces present in more confined spaces like the small intestine^9^. In addition, they also risk getting stuck and trapped in more constrained and tortuous environments as the large amount of contact friction prevents effective control, restricting their maneuverability^10–14^.

In contrast, spring-like origami crawlers have demonstrated the ability to achieve robust locomotion in confined narrow spaces due to their ability to overcome the environmental resistance and contact pressure imposed by small lumen diameters and the presence of constant muscle contractions^10,15,16^. However, such origami crawlers are unimodally stable and inherently compliant for continuous deformation, and often employ the unimodal deformation conferred by their folding DoFs for locomotion^15,17,18^. This restricts them to simple non-load bearing medical functions including simple cargo transport, passive drug delivery via diffusion and dissolution and battery removal using an on-board magnet^15,17^, that do not require additional DoFs to execute.

One approach to extend the functionalities of origami crawlers is to enable a separation of the folding DoFs of the mechanism, such that a subset of these folding DoFs can be utilized for locomotion, while the remaining can be used for functionalities. We hypothesize that imparting bistability into the underlying crease pattern of the origami mechanism can enable the presence of energy-dependent hierarchical DoFs that are separated by energy barriers conferred by the bistable regime^19–21^.

Here, we propose a miniature magnetically actuated bistable origami crawler of size 30 mm in length and 9 mm in diameter by introducing degree-four vertices into the existing Tachi Miura crease pattern. The bistable origami crawler can be compressed to fit into a size 000 capsule and can shape-morph to access two separate regimes of folding DoFs for locomotion and load-bearing functionalities. Specifically, the origami crawler utilizes the folding DoFs in one regime for continuous inchworm-inspired crawling deformation for robust steering and locomotion (crawling velocity up to 1.86 mm s^−1^) on porcine intestinal tissue and in high contact friction environments, such as when being sandwiched between two ecoflex layers. We demonstrated the bistable origami crawler’s ability to reconfigure and access the other bistable regime to adjust its compliance and carry out load-bearing tasks like microneedles insertion. The results show the potential of the bistable origami crawler to be used for load-bearing applications in narrow confined spaces.

## Results and discussion

### Magnetically actuated bistable V-fold mechanism

Among the various origami patterns, the Kresling pattern stood out as a commonly used building block for realizing origami crawlers via extension and compression under torque^15,22^. The bistability of the Kresling mechanism can be attributed to the stretching of panels^23^, which can be accessed via a coupled rotational and axial DoF. As a result, the deformation of bistable origami cylinders using the Kresling as the fundamental origami unit often involves actuation methods that impart rotational torques in order to fully extend the Kresling units to access the bistable regime^21,24,25^. However, actuation schemes for multimodal shape-morphing deformation of compliant crawlers using magnetic actuation often involves linear extension and compression via out-of-plane bending of the backbone^26–33^. Using actuation methods to achieve multimodal deformation of Kresling-based origami crawlers would necessitate the integration of independent additional actuators to impart rotational torque to access the bistable regime, greatly complicating the control and fabrication of the mechanism, especially since the same magnetic field is usually used to achieve parallel and coordinate control of multiple folds to achieve the desired DoFs in origami mechanisms^2^. In addition, the coupled rotation and linear DoF might not be desirable in conditions where the orientation of the deployed facets of the origami mechanism matters.

**Fig. 1:**
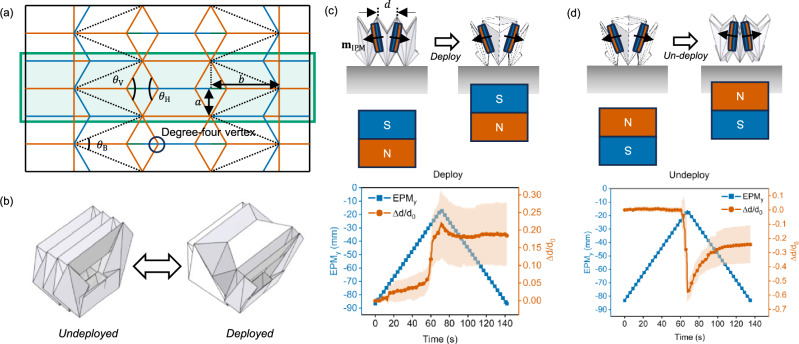
Magnetically actuated bistable V-fold mechanism. **a** Introduction of degree-four vertex into the V-fold origami crease pattern. The crease pattern is of the mechanism when it is deployed. A tessellated unit is boxed in green. ${{{\boldsymbol{\theta }}}}_{{{\bf{V}}}}$, ${{{\boldsymbol{\theta }}}}_{{{\bf{H}}}}$, ${{{\boldsymbol{\theta }}}}_{{{\bf{B}}}}$, ${{\boldsymbol{a}}}$ and ${{\boldsymbol{b}}}$ refers to the V-fold angle, the hidden angle, the bending angle, the half-length and height of the triangulated V-fold in each unit of the bistable V-origami crease pattern, respectively. **b** Bending-induced snap-through bistability of the corresponding folded mechanism. **c** Deployment of the bistable V-fold unit can be achieved via unfolding and consequently inversion of the degree-four vertex when the south pole of the external permanent magnet (EPM) is brought close to the mechanism. **d** Undeployment of the bistable V-fold unit can be achieved via folding and consequently re-inversion of the degree-four vertex when the north pole of the EPM is brought close to the mechanism. In both graphs, three samples were used and the standard error bars are calculated using standard deviation in excel.

An alternative to the Kresling is the Miura-Ori pattern^33–35^ which can exhibit tunable bistability under linear extension and compression^36^. In contrast to the Kresling, the bistability of the Miura Ori can be attributed to the bending of compliant panels^23^, where snap-through bistability is exhibited around the point of maximum length under linear extension and compression^37^. In our previous work, we modified the existing Tachi Miura crease pattern and defined the hidden DoFs by placing creases where bending occurs^20^. We introduced additional mountain creases into the desired deployable facet to create degree-four vertices that can be locally inflected under displacement to enable snap-through bistability (Fig. 1a, b), such that out-of-plane bending of the mechanism can be used to access the bistable regime. The modified bistable V-fold crease pattern can be characterized by the following three parameters: the V-fold angle (${\theta }_{{{\rm{V}}}}$), the hidden angle (${\theta }_{{{\rm{H}}}}$), and the bending angle (${\theta }_{{{\rm{B}}}}$). By choosing the appropriate values of these three parameters, we can tailor the geometry and properties of the folded bistable V-fold mechanism (Refer to Supplementary Note 1 and Supplementary Figs. S1–S3 for more information on the design considerations for each parameter). We standardized the dimensions of the three parameters as follows: ${\theta }_{{{\rm{V}}}}={\theta }_{{{\rm{H}}}}=\,120$°, and ${\theta }_{{{\rm{B}}}}=45$°.

We fabricated a miniature bistable V-fold module that consists of three units of the tessellated bistable V-fold pattern using 50-μm-thick Polyethylene Terephthalate (PET) sheets. The dimension of the crease pattern (13.5 mm by 23 mm) was chosen such that in the folded configuration, the module could fit comfortably into a size 000 capsule (see Supplementary Note 2 and Supplementary Fig. S4 for more details on fabrication). To enable untethered folding and bending of the bistable V-fold module, we attached an internal permanent magnet (IPM) to each of the side units such that their magnetic moments ${{{\bf{m}}}}_{{{\rm{IPM}}}}$ aligned along the axial direction (the $x$-axis according to our nomenclature) and mirrored each other. Under the influence of an external magnetic field generated by an N52 permanent magnet (side 25 mm), the central unit will undergo folding/unfolding and consequently deployment/undeployment (Refer to Supplementary Note 3 and Supplementary Fig. S5 for characterization of the EPM).

Based on the orientation of the IPMs in the bistable V-fold origami mechanism, positioning the south pole of the EPM close to the base of the origami module (increasing ${{EPM}}_{y}$) would cause “outwards” rotational moment of both IPMs due to the imposed forces ${{\bf{f}}}$ and torques ${{\boldsymbol{\tau }}}$ (Refer to Supplementary Note 3, Supplementary Fig. S6 for experimental setup, and Supplementary Figs. S8 and S9 for details on the principles behind magnetic actuation). This causes the central crease between the two degree-four vertices to unfold and eventually deploy under a sufficiently strong magnetic field, as observed by the sudden increase in the normalized deformation of the origami mechanism ($\Delta d/{d}_{0}$) in Fig. 1c. From experimental data (Supplementary Fig. S7a and Supplementary Table S1), the average distance between the center of the EPM to the origami mechanism required for deployment was estimated to be ${\sim} 27.2\pm 1.0\,{{\rm{mm}}}$, which corresponds to magnetic field strength of around ${\sim} 72.2\pm 7.8\,{{\rm{mT}}}$. Further increasing the magnetic field strength by bringing the south pole of the EPM closer led to further unfolding of the central crease until the maximum structural limit was reached. When the south pole of the EPM is brought away, the degree-four vertex remains inverted and the bistable V-fold remains in its deployed state, which is indicative of bistability.

Conversely, positioning the north pole of the EPM close to the base of the origami module would cause “inward” rotational moments of both IPMs, causing the central cease to fold and eventually buckle. This causes the module to undeploy if it was previously deployed under a sufficiently strong magnetic field, indicated by the sudden decrease in the normalized deformation of the origami mechanism ($\Delta d/{d}_{0}$) as observed in Fig. 1d. From experimental data (Supplementary Fig. S7b and Supplementary Table S2), the average distance between the center of the EPM to the origami mechanism required for undeployment was estimated to be ${\sim} 18.8\pm 2.1\,{{\rm{mm}}}$, which corresponds to magnetic field strength of around ${\sim} 152.0\pm 46.6\,{{\rm{mT}}}$ (average of three samples).

The sudden change in deformation values in Fig. 1c, d suggests a rapid snap-through of the origami mechanism when it switches between its bistable configurations (Supplementary Movie S1). This is attributed to the inversion and re-inversion of the degree-four vertex resulting from the bending-induced tensional stresses imposed on the central crease because of the rotation of the IPMs under the influence of an external magnetic field. We further observed that the deployment of the origami mechanism resulted in a natural curvature due to the breaking of folding symmetry as observed in Supplementary Fig. S3. Hence, the rapid snap-through behavior observed in the module under bending stresses along its natural curvature axis was likely attributed to this asymmetricity.

### Magnetically actuated bistable origami crawler

**Fig. 2:**
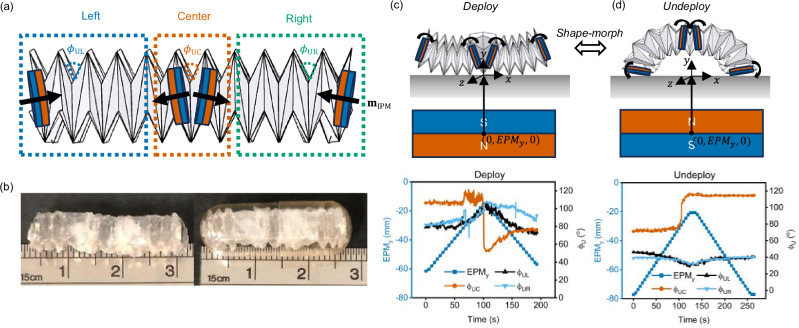
Bistable origami crawler. **a** The origami crawler consists of three segments (left, center, and right), and the four internal permanent magnets (IPMs) are positioned and oriented in the crawler backbone as shown. The folding angles of the central unit of the left (${{{\boldsymbol{\phi }}}}_{{{\bf{UL}}}}$), center (${{{\boldsymbol{\phi }}}}_{{{\bf{UC}}}}$), and right (${{{\boldsymbol{\phi }}}}_{{{\bf{UR}}}}$) segments were used to characterize the shape of the origami crawler. **b** The length and diameter of the origami crawler at rest was roughly 30 mm and 9 mm, respectively. The origami crawler could also be compressed to fit inside a size 000 capsule. **c** Deployment of the origami crawler can be achieved via unfolding and consequently inversion of the degree-four vertex when the south pole of the external permanent magnet (EPM) is brought close to the mechanism. **d** Undeployment of the bistable V-fold unit can be achieved via folding and consequently re-inversion of the degree-four vertex when the north pole of the EPM is brought close to the mechanism.

We fabricated the origami crawler as three segments (left, center, right) with four IPMs (Fig. 2a). The center segment consisted of a single magnetically actuated bistable V-fold origami module comprising of three units of tessellated bistable V-fold pattern as in the previous section. Each of the left and right segments consisted of five units of tessellated bistable V-folds, with an IPM attached onto one of the ends. In this case, the left and right segments were responsible for generating the friction anisotropy required for directional locomotion while the central segment actively contracted and extended (via folding and unfolding) to allow deformation of the origami crawler in its undeployed state (see the Supplementary Note 4 for more details on design and fabrication of the origami crawler). The fabricated origami crawler was 30 mm and 9 mm in length and diameter at rest, respectively, and could be compressed to fit inside a size 000 capsule of 22 mm and 9.5 mm in length and diameter, respectively (Fig. 2b). Inclusive of the IPMs, the origami crawler weighed 0.78 g in total.

The magnetic pattern of the origami crawler was designed with the following functionality in mind: (i) the central unit is undeployed during locomotion to maximize the flexibility of the origami crawler, and (ii) the central unit is deployed when the origami crawler wants to execute load-bearing applications (Fig. 2c). For analysis purposes, we characterized the shape of the origami crawler using the following three parameters: the folding angle of the central unit of the left segment (${\phi }_{{{\rm{UL}}}}$), the folding angle of the central unit of the right segment (${\phi }_{{{\rm{UR}}}}$), and the folding angle of the central unit (${\phi }_{{{\rm{UC}}}}$) of the origami crawler (Fig. 2a). The folding angle of the origami crawler can be calculated based on simple geometry (see Supplementary Note 5 and Supplementary Fig. S11 for calculations of folding angle).

For the given magnetic pattern of the origami crawler, positioning the south pole of the EPM close to the base of the central segment of the origami crawler created an “outwards” rotational moment of the IPMs located on both ends of the central segment due to the imposed forces and torques, causing the central crease between the two degree-four vertices to unfold (increase in ${\phi }_{{{\rm{UC}}}}$), and eventually deploy under a sufficiently strong magnetic field (Fig. 2c). In addition, the generated magnetic field simultaneously caused an “inwards” rotation of the IPMs on the ends of the left and right segment, causing the V-folds to undergo folding (${\phi }_{{{\rm{UL}}}}$ and ${\phi }_{{{\rm{UR}}}}$ decrease), resulting in a compression of the left and right segments. This caused the centrally deployed unit to be anchored while the ends of the origami crawler were lifted. From Fig. 2c, We observed that the resting angle of the central segment was naturally larger than the left and right segments due to repulsion between the two central IPMs. When the south pole of the EPM was brought closer to the origami crawler, the central segment unfolded (increasing ${\phi }_{{{\rm{UC}}}}$) while the left and right segments folded (decreasing ${\phi }_{{{\rm{UL}}}}$ and ${\phi }_{{{\rm{UR}}}}$). The central segment of the crawler deployed (sudden jump in ${\phi }_{{{\rm{UC}}}}$) when the EPM was positioned ~29 mm away from the origami crawler, corresponding to a magnetic field strength of ~63.0 mT. Further increasing the magnetic field strength by bringing the south pole of the EPM closer led to further increase in ${\phi }_{{{\rm{UC}}}}$ until the central segment reaches its structural limit. The central segment remained deployed even after bringing the south pole of the EPM away (${\phi }_{{{\rm{UC}}}}$ remained somewhat constant), while the left and right segments unfolded (increasing ${\phi }_{{{\rm{UL}}}}$ and ${\phi }_{{{\rm{UR}}}}$) due to the inherent folding stiffness of the creases until they returned to their resting angle.

In contrast, positioning the north pole of the EPM close to the base of the central segment of the origami crawler will cause an “inwards” rotational moment of the IPMs located on both ends of the central segment due to the imposed forces and torques. When the EPM was brought sufficiently close (~15 mm), the central segment of the crawler un-deploys (sudden drop in ${\phi }_{{{\rm{UC}}}}$), which corresponded to a much stronger magnetic field strength of ~272.2 mT than that needed to deploy the central segment (Fig. 2d). Under the same changing magnetic field, the V-folds of the left and right segments underwent unfolding (${\phi }_{{{\rm{UL}}}}$ and ${\phi }_{{{\rm{UR}}}}$ increase), resulting in an extension of both segments. This caused the central undeployed unit to be lifted while the two ends of the origami crawler anchored onto the surface, giving rise to a shape similar to an “omega”. Bringing the north pole of the EPM away resulted in the unfolding of the central segment of the origami module (increasing ${\phi }_{{{\rm{UC}}}}$), while the left and right segments folded (decreasing ${\phi }_{{{\rm{UL}}}}$ and ${\phi }_{{{\rm{UR}}}}$) due to the inherent folding stiffness of the crease until they returned to their resting angle. For the experimental setup, refer to the Supplementary Note 5 and Supplementary Fig. S10.

### Bidirectional locomotion of the origami crawler

**Fig. 3:**
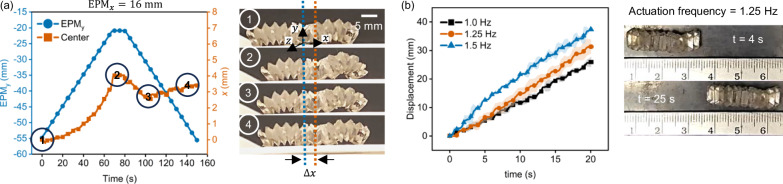
Directional locomotion of the origami crawler. **a** Placing the north pole of the external permanent magnet (EPM) sufficiently far ahead from the center of the origami crawler (${{{\boldsymbol{EPM}}}}_{{{\boldsymbol{x}}}}$) resulted in net forward displacement ${{\boldsymbol{\Delta }}}{{\boldsymbol{x}}}$, which can be used to enable bi-directional locomotion. **b** Distance traveled by the origami spring over time when actuated under different frequencies. In general, the faster the actuation frequency, the faster the locomotion speed.

Several origami crawlers in literature incorporated friction-anisotropic structures such as directional feet to convert the deformations of the origami crawler into translational motion^15,38^, While this approach enables directional locomotion under a uniform unchanging field, it did not allow for bidirectional locomotion. An alternative approach involves generating frictional anisotropy by actively modulating the curvature of the origami mechanism to achieve asymmetric deformation^27,39^. This asymmetric shape deformation can be achieved by varying the magnitude and alignment of the magnetic field via either generation of magnetic gradient-based forces, or generation of an asymmetric magnetic field about an axis to produce displacement in the desired direction. Since the source of our magnetic field was a permanent magnet, the generated field was inherently non-uniform and gradient-based, which could be used to create asymmetric magnetic fields to modulate the degree by which either end of the origami crawler anchored onto the surface to create the frictional anisotropy required for directional locomotion. More specifically, by positioning the EPM closer to one of the end segments of the crawler when it is undergoing locomotion in its undeployed state, the corresponding IPM will experience a stronger magnetic field and consequently magnetic force and torque. This caused the selected IPM(s) to rotate more than the others, which consequently allowed us to modulate the degree of curvature that each segment of the origami crawler underwent, biasing the direction in which it moved (Fig. 3a). When the EPM is brought closer to the origami crawler, the front end of the origami crawler serves as an anchor that enables the origami crawler to pull its other end forward to generate directional displacement (①–②). When the EPM was moved away from the origami crawler, the inherent stiffness and elasticity of the origami crawler caused the front segment to straighten and return to its resting position. During this period, the back segment of the origami crawler acted as an anchor, allowing the front end to slide forward (②–④). More information on the generation of friction anisotropy via modulation of curvature can be found in Supplementary Note 5 and Supplementary Figs. S1 and S13. From experiments, we observed that the optimal distance between the EPM and the center of the origami for directional locomotion is ~16 mm. When the EPM was not positioned sufficiently ahead of the center of the origami crawler, insufficient net displacement would be generated. Conversely, positioning the EPM too far ahead led to undesired slippage (Supplementary Note 5 and Supplementary Fig. S14).

Depending on the actuation frequency of the EPM, the crawling speed of the origami crawler can be controlled (Fig. 3b). As expected, the origami crawler was able to travel faster when actuated at a higher frequency, demonstrating faster locomotion speed. The average traveling speed of the origami crawler under the actuation frequencies of 1.0 Hz, 1.25 Hz, and 1.5 Hz was calculated to be ${\sim} 1.29\pm 0.096$ mm s^−1^ (${\sim} 0.0585\pm 0.005$ bodylength s^−1^), ${\sim} 1.56\pm 0.129$ mm s^−1^ (${\sim} 0.0708\pm 0.006$ bodylength s^−1^), and ${\sim} 1.86\pm 0.023$ mm s^−1^ (${\sim} 0.0844\pm 0.001$ bodylength s^−1^), respectively (refer to Supplementary Note 5, Supplementary Fig. S15 and Supplementary Table S3). A comparison between the normalized crawling speed of the bistable origami crawler against other crawlers in the literature can be found in Table 1.

**Table 1:** Comparison of traveling speed of bistable origami crawler against literature

Actuation principle	Locomotion achieved	Normalized velocity to 3 s.f. (bodylength/s)	Ref.
Magnetic	In-plane	0.0294	^50^
Light	In-plane	0.0345	^51^
Motor	In-plane	0.0204	^18^
Motors	In-plane	0.106	^52^
SMA	In-plane	0.00308	^38^
Magnetic	Out-of-plane	0.0418	^53^
Magnetic	Out-of-plane	0.00833	^27^
Magnetic	Out-of-plane	2.31	^54^
Magnetic	Out-of-plane	2.87	^55^
SMA	Out-of-plane	0.0667	^56^
Magnetic	In-plane	0.66	^15^
Thermal	Out-of-plane	0.012	^57^
Magnetic	Out-of-plane	0.084389	Our work

### Navigation in confined environments

**Fig. 4:**
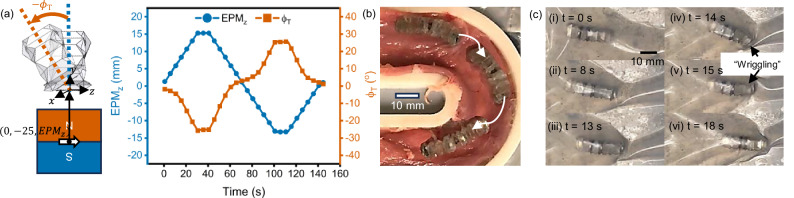
Navigation of the origami crawler. **a** Tilting of the origami crawler can be achieved by displacing the north pole of the external permanent magnet (EPM) along the ${{\boldsymbol{z}}}$-axis. The further the EPM was along the ${{\boldsymbol{z}}}$-axis relative to the center of the origami crawler, the greater the tilt as measured by the tilt angle (${{{\boldsymbol{\phi }}}}_{{{\bf{T}}}}$). **b** Steering of the origami crawler via counter-steering to navigate curvatures on porcine tissue. **c** The origami crawler demonstrated robust locomotion in confined spaces. In the case of encountering collapsed regions, the origami spring could “wriggle” to make space to crawl through.

We further observed that we could tilt the origami crawler by positioning the north pole of the EPM away from the origami crawler along the $z$-axis (refer to Supplementary Note 5 and Supplementary Fig. S16 for experimental setup). Positioning the EPM along the positive $z$-axis caused the origami crawler to tilt counterclockwise towards the opposite direction (negative ${\phi }_{{{\rm{T}}}}$) as shown in Fig. 4a. Conversely, positioning the EPM along the negative $z$-axis caused the origami crawler to tilt clockwise in the positive $z$-axis. As the EPM was positioned further along the $z$-axis relative to the origami crawler, the origami crawler tilted more, as evidenced by the increasing absolute magnitude of the tilt angle (${\phi }_{{{\rm{T}}}}$) of the origami crawler as the EPM was positioned further from the crawler. This tilting could be used to achieve steering of the origami crawler during directional locomotion. In addition to positioning the EPM in the positive $x$-axis from the center of the origami crawler, a slight displacement in the $z$-axis caused the origami crawler to tilt while anchoring in the direction of the EPM. As the origami crawler tilted, one side of the crawler experiences greater friction causing a slight rotation, similar to the concept of “counter-steering” employed in motorcycles. To demonstrate the steering capabilities of the origami crawler, we actuated the origami crawler on porcine small intestine tissue and navigated it around a curvature (Fig. 4b, Supplementary Movie S2).

We tested the ability of the origami spring to traverse in a confined environment by sandwiching it between two Ecoflex 00-10 layers, similar to what Qiji et al^15^ had done in their work (Supplementary Movie S3). From Fig. 4c, we observed that the origami spring was capable of robust locomotion in confined environments despite the presence of great environmental resistance (Fig. 4c(i)–(ii)) In addition, upon encountering collapsed regions (Fig. 4c(iii)), we noticed that rapidly moving the north pole of the EPM back and forth along the $z$-axis caused the origami spring to bend from side to side quickly, in a manner that was similar to “wriggling” (Fig. 4c(iv)–(v)). This wriggling could distend the collapsed Ecoflex film to create space for the origami spring to crawl through (Fig. 4c(vi)). To further verify the capabilities of the origami crawler in endoluminal applications, we actuated the origami crawler through an enclosed tubular porcine small intestine sample and observed that the crawler was able to successfully navigate through the entire length of the small intestine segment (Supplementary Movie S4).

### Microneedles delivery and insertion

Untethered ingestible origami mechanisms capable of robust locomotion in confined spaces and reconfiguration for the execution of functions can have great potential for medical interventions in the GI tract. These origami mechanisms can have the ability to non-invasively access and navigate in difficult-to-reach, narrow areas inside our body, as well as being able to execute forces or bear loads during interactions with tissues. For the proposed bistable origami crawler, the inflection of creases into pseudo-facets has promising applications in biomedical applications such as a safety mechanism to prevent accidental undeployment of housed components during transport^6–8^, or even to coat functional components^40^ or integrate mucosa-interfacing electronics and structures^41^ onto the facets that can be exposed on-demand during deployment. One particular biomedical application that can benefit from this is the delivery of microneedles in confined environments like the small intestine. Microneedles have a wide range of biomedical applications, including but not limited to: biologics and drugs delivery^42^, electrical stimulation^43^, biopotential and deep tissue sensing^44^, anchoring^41^, and even act as waveguides for optical illumination^44^. Hence, ingestible devices that can enable rapid and site-specific delivery of microneedles are desirable for targeted diagnostic and therapeutic interventions^45^.

**Fig. 5:**
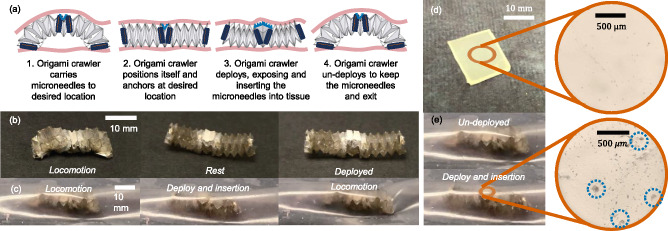
Bistable origami crawler for delivery and insertion of microneedles. **a** Concept of the origami crawler for microneedles delivery. **b** Adhering the microneedles onto the central segment of the origami crawler. The origami crawler was able to adopt the deployed and undeployed state even when the microneedles were attached. **c** Attachment of the microneedles onto the origami crawler did not hinder the shape-morphing ability of the origami crawler in confined environments. **d** Topology of an un-punctured Ecoflex film. **e** Ecoflex film punctured by microneedles inserted by the origami crawler. Identified puncture holes are in green circles.

For the specific application of microneedle delivery and insertion in confined spaces, we proposed the following concept as shown in Fig. 5a. As a proof of concept, we attached hyaluronic acid microneedle patches (CASMA Acne Microcrystal Patches) to the central segment of the origami crawler. Figure 5b shows the microneedle-carrying origami crawler in the rest, undeployed locomotion and deployed state, respectively. We then demonstrated the ability of the origami spring to deliver and insert the microneedles in the same confined Ecoflex sandwich setup as before (Fig. 5c, Supplementary Movie S5). As observed, the addition of the microneedles did not hinder the ability of the origami spring to locomote and reconfigure its shape. To verify the potential of the origami crawler in microneedle insertion applications, we view the topology of the punctured Ecoflex film to confirm the presence of visible puncture marks under the microscope (Binocular 50000, Tikuo Inc., China) as shown in Fig. 5e in comparison to the non-punctured ecoflex film (Fig. 5d). More information can be found in the Supplementary Note 6 and Supplementary Fig. S17.

### Conclusion

In this work, we employed a modified Tachi-MIura origami crease pattern as the fundamental unit for the design and fabrication of a bistable origami crawler. By integrating permanent magnets into the architecture of the origami crawler, we were able to achieve untethered shape-morphing and reconfiguration between two different states under the influence of a changing magnetic field. In each state, the origami crawler is capable of folding and unfolding for either active robust locomotion in confined environments, or load-bearing applications. We verified the capability of the origami crawler for robust locomotion, steering, and navigation when sandwiched between two Ecoflex-layers, as well as in an ex vivo porcine small intestine. To illustrate the benefits of conferring bistability to origami mechanisms, we demonstrated how the origami crawler can reconfigure and access a separate regime when the polarity of the applied magnetic field is changed. In this configuration, the origami mechanism is capable of load-bearing applications, such as microneedle insertion, as demonstrated in this work. Moving forward, the origami crawler can be encapsulated in biocompatible elastomeric materials to minimize injury risks associated with detachment of IPMs and the sharp folds of the folded origami crawler. While our work mainly focused on endoluminal confined applications, we believe that our work can lay a foundation for how integration bistability into origami mechanisms can enable greater functionalities and capabilities in other applications. For example, beyond microneedle insertion and delivery, other deployable and load-bearing applications such as fluid collection^46^, stents^47^, and airway support mechanisms^48^ may potentially be integrated. In addition, other actuation methods (e.g., pneumatic) may also be integrated with the bistable origami mechanisms designed for other applications that can benefit from its reconfigurability, such as in space deployment structures^37^ or as temporary shelters^49^.

## Methods

### Fabrication of the bistable V-fold mechanism

The crease pattern of the origami mechanism was folded using laser-cut 50-μm-think Polyethylene Terephthalate (PET) sheets. Three units of bistable origami V-fold were then folded and the glue tabs are adhered together using silicone adhesive. An N35 permanent disc magnet (5 mm diameter, 1 mm thickness) was attached into each end of the folded mechanism using food grade silicone adhesive.

### Fabrication of the bistable origami crawler

The side segments of the origami crawler was fabricated from 50-μm-thick PET sheet via laser cutting and folding. For simplicity, we chose the same set of parameters for the side segments as that of the bistable V-fold (i.e., ${\theta }_{{{\rm{V}}}}={\theta }_{{{\rm{H}}}}=\,120$°, and ${\theta }_{{{\rm{B}}}}=45$°). The number of units in the left and right segments were chosen to optimize the deformation of the crawler to achieve the desired shapes. An N35 permanent disc magnet (5 mm diameter, 1 mm thickness) was adhered to one external ends of the origami crawler in the desired orientation. Food grade silicone adhesive was used to adhere the segments and IPMs.

### Magnetic actuation

We used an N52 neodymium-iron-boron (NdFeB) alloy cube magnet (side 25 mm) from Titan™ Magnetics as the EPM, while N35 permanent disc magnets (5 mm diameter, 1 mm thickness, purchased from Titan™ Magnetics) were used as the IPMs.

More details about the design, fabrication and experimental setup for the characterization of the origami samples are provided in the Supplementary materials.

## Supplementary information

Peer Review file

Supplementary Material

Description of Additional Supplementary Files

Movie S1

Movie S2

Movie S3

Movie S4

Movie S5

Supplementary Data 1

## Data Availability

The source data needed to evaluate the conclusions of the paper and for the figures presented in this paper can be found in the main manuscript and the supplementary information. Additional primary data that support the findings of this study are available from the corresponding author upon request.
