# Supplementary material for: Untethered bistable origami crawler for confined applications
Source: Commun Eng. 2024 Oct 30;3:150. doi: 10.1038/s44172-024-00294-1 (PMC11525557; doi:10.1038/s44172-024-00294-1)
Supplement: Supplementary file 2 — Supplementary Material [file 44172_2024_294_MOESM2_ESM.pdf]

Untethered bistable origami crawler for confined applications

*Catherine Jiayi Cai[1,2,3,#], Hui Huang[2,4], and Hongliang Ren[1,3]**

1. Department of Biomedical Engineering, National University of Singapore, Singapore 117575, Singapore;
2. Singapore Institute of Manufacturing Technology, Agency for Science, Technology and Research (A*STAR), 5 Cleantech Loop, Singapore 636732;
3. Department of Electronic Engineering, Faculty of Engineering, The Chinese University of Hong Kong, Hong Kong, China;
4. Engineering Cluster, Singapore Institute of Technology, 10 Dover Drive Singapore 138683;

# Present address: Department of Mechanical Engineering, National University of Singapore, Singapore 117575, Singapore

* Corresponding to hlren@ieee.org

This PDF file includes:

Supplementary Notes

Supplementary Figures S1 to S17

Supplementary Tables. S1 to S3

Legends for Supplementary Movies S1 to S5

Supplementary Data

Supplementary References

Other Supplementary Material for this manuscript includes the following:

Supplementary Movies S1 to S5

Supplementary Note

<u>Supplementary Note 1: Crease pattern parameters in the design of the Bistable V-fold unit</u>

Choice of V-fold angle ($\boldsymbol{\theta_{\mathrm{V}}}$). The Tachi Miura crease pattern consists of iterative layers that each consists of mirroring V-folds that globally fold to form a cylindrical span. According to Jackson [1], the V-folds are characterized by their distinctive 'V' shape, in which three mountain folds and one valley fold (or one mountain fold and three valley folds) meet at a node. The individual V-folds achieve targeted folding angles by kinematically coupling with other folding angles [2]. Depending on the angle between the strokes of each V (V-fold angle, θ_{V}), one can open or close the span [1]. Fig. S1 (a) shows the generic crease pattern of a four-sided V-fold cylindrical bellows with a θ_{V} of 90° (also commonly known as the accordion bellow), and the folded counterpart generated using an online origami simulator [3]. Similar to the Kresling, V-fold cylinders are flat-foldable but not rigid-foldable. This can be attributed to Conelly's bellows theorem [4], [5]. Unlike the Kresling however, the cylindrical V-fold pattern is able to generate purely translational motion.

The folding angle of the crease pattern can greatly influence the geometry of the folded origami cylinder. A larger folding angle ($\theta_{\mathrm{V}} \geq 90°$) created a smaller closing angle ($\phi_{\mathrm{V}} \leq 90°$) to allow for a greater span, resulting in a more rounded and cylindrical tubular structure with polygonal faces. In contrast, a smaller folding angle ($\theta_{\mathrm{V}} \leq 90°$)) created a larger closing angle ($\phi_{\mathrm{V}} \geq 90°$), resulting in a more angled and compact box-like structure. Fig. S1 (b) shows the crease patterns of the V-fold origami for θ_{V} = 60°, 90° and 120°, along with the simulated folded counterparts. Due to space constraints in confined regions, we preferred larger closing angles in the design of the origami crawler due to its compactness. However, at larger θ_{V}, the folding of the origami pattern risked collision within each layer. To avoid this, we modified the crease pattern by halving the crease pattern and included straight folds that could be folded in and glued together to create an enclosed structure (Fig. S1 (c)).

Choice of Hidden angle ($\boldsymbol{\theta_{\mathrm{H}}}$). As the V-fold pattern was non-rigid foldable like the Kresling, V-fold cylinders were rarely fully extended to avoid over-straining the vertices which could cause the creases to invert, resulting in undesirable buckling and deformation [6]. As such, V-fold cylinders were often designed to be unimodally stable and inherently compliant. Silverberg et al [7], there existed a hierarchy of degree of freedom (DoF) that each have their own associated energy scale. Elaborating on this, they demonstrated how non-rigid foldable origami exhibits hidden bending DoFs that were separated from the crease DoF by an energy gap, giving rise to a critical bifurcation between mono-stability and bistability that was geometrically driven.

Upon unfolding a module of the modified V-fold origami pattern, we observed that beyond a certain unfolding angle, the facets began to bend during the unfolding process, eventually causing the valley crease to invert and access a different configuration (Fig. S2 (a)). In addition, the structure was able to hold this configuration even in the absence of external force, indicating that the origami module had reconfigured into a different stable state. Silverberg et al [7] referred to this

phenomenon as an energy-scale dependent DoF that resulted from the geometry of creased sheets in constraint-based compliant materials.

Similar to Melancon et al [8], we further modified the crease pattern of the V-fold origami by placing mountain creases of an angle (θ_{H}) where the observed outward bending of the facets occurred to invert the valley crease, creating a degree-four vertex (Fig. 1 (a)). Under sufficient external forces, the creases around these vertices were observed to inflect locally, causing the V-fold origami to deploy into the other configuration. The deployed configurations of the folded prototypes with defined and undefined θ_{H} are shown in Fig. S2 (b)

Choice of Bending angle ($\boldsymbol{\theta}_{\mathbf{B}}$). Upon deployment of the bistable V-fold module, it adopted a rigid curved configuration with a tunable bending radius (ϕ_{B}) governed by the ratio of the of the height (b) and half-length (a) of the triangulated V-fold in each unit of the bistable V-origami crease pattern (Fig S3 (b)). From geometry, the angle θ_{B} was calculated to be:

$$\theta_{\mathrm{B}} = 2 * \tan^{-1}\left(\frac{a}{b}\right) \tag{1}$$

We further measured the bending radius ϕ_{B} of the folded deployed bistable V-fold origami module corresponding to their respective θ_{B} and best-fitted the results in Origin (Fig. S3 (c)). We observed that the relationship between the two could be approximated as such:

$$\phi_{\mathrm{B}} = 1.22 * \theta_{\mathrm{B}} - 12.2 \tag{2}$$

When combinations of individual bistable V-fold modules were deployed in an aggregated bistable V-fold origami, the resulting structure was compliant around a bending axis up to a specific angle, at which it was unable to bend further due to the inherent structure (Fig S3 (b)). The minimum bending angle ($\phi_{\mathrm{B\_total}}$) that the structure can achieve was approximated to be the sum of the bending radius ϕ_{B} of the individual deployed V-fold origami modules.

In this work, we chose $\theta_{\mathrm{B}} = 45°$ as an arbitrary value. Depending on the desired bending radius of the application, θ_{B} can be tuned accordingly.

Supplementary Note 2: Fabrication of the bistable V-fold mechanism

V-fold Origami. The crease pattern of the origami mechanism was drawn in Inkscape with a line thickness of 0.1 mm, and the crease lines as a series of small holes in a straight line. We used $50\,\mu\mathrm{m}$ think Polyethylene Terephthalate (PET) sheets (purchased from Jubang plastic material, Inc.) for the folding of the origami mechanisms in this work. The material then defined in the lasercut software (xTool Creative Space) was "Brown Kraft Paper" of 0.2 mm thickness (Fig. S4 (a)). The laser parameters were set to “cut” with a flat laser, with the power and speed set at 40% and 15 respectively for a total of 1 pass (Fig. S4 (b)). Three units of bistable origami V-fold is then folded and the glue tabs are adhered together using silicone adhesive (Cutorin 4672-B food grade silicone). To maximize the translation of the torque that the IPMs experience under the influence of the EPM to the folding and unfolding of

the central crease, the side units housing the IPMs are clamped to the central unit using silicone adhesive to remove the folding DoFs of the side units.

Internal Permanent Magnets (IPMs). To a three-unit bistable origami V-fold mechanism, we tucked and attached an N35 permanent disc magnet (5 mm diameter, 1 mm thickness, purchased from Titan™ Magnetics) into each end of the folded mechanism (in each end unit of the crease pattern) such that their magnetic moments mirrored each other (Fig. S4 (b)). The disc magnets were chosen because their flatness enabled them to integrate well with the flat foldable nature of the V-fold origami. While the diameter of the IPM was arbitrary, we chose the maximum size that could fit into the origami as a larger magnet was usually associated with greater magnetic flux and hence could be actuated using weaker magnetic fields [9]. Food grade silicone adhesive was used to adhere the IPMs to the V-fold mechanism.

Supplementary Note 3: Magnetic Actuation of the bistable V-fold mechanism

External Permanent Magnet (EPM). We used an N52 neodymium-iron-boron (NdFeB) alloy cube magnet (side 25 mm) from Titan™ Magnetics as the EPM. Similar to the IPMs, the size and shape of the EPM used can be arbitrary, as long as it was able to generate sufficiently strong enough magnetic fields for the intended purpose.

Principles of magnetic actuation. Fig. S5 (a) shows a representation of a magnetic dipole (of magnetic moment $\mathbf{m}$) generating magnetic field lines that are radially symmetrical about its magnetization direction. According to [10], the magnetic field $\mathbf{B}$ generated tangent to the magnetic field lines by a magnetic dipole (located at $\mathbf{P}_\mathrm{m}$) on a region of interest (located at $\mathbf{P}_\mathrm{b}$) can be approximated by the dipole model as:

$$\mathbf{B}\{\mathbf{r},\mathbf{m}\} = \frac{\mu_0}{4\pi|\mathbf{r}|^3}(3\hat{\mathbf{r}}\hat{\mathbf{r}}^\mathrm{T} - \mathbf{I})\mathbf{m} \tag{3}$$

where $\mu_0 = 4\pi \times 10^{-7}\ \mathrm{T}\cdot\mathrm{m}\cdot\mathrm{A}^{-1}$ is the permeability of free space, $\mathbf{r} = \mathbf{P}_\mathrm{b} - \mathbf{P}_\mathrm{m}$, and $\mathbf{I}$ is the identity matrix.

Upon interaction between the permanent magnets, the magnetic field from the EPM generates forces and torques that, if strong enough to overcome the inherent folding stiffness of the creases, can induce unfolding and folding and consequently deployment and Undeployment respectively of the bistable origami mechanism respectively. The resulting force $(\mathbf{f})$ and torque $(\boldsymbol{\tau})$ imposed on an IPM (and consequently on the origami crawler backbone) can be calculated using the following equations:

$$\mathbf{f}_i = (\nabla\mathbf{B}_i \cdot \mathbf{m}_i) \tag{4}$$

$$\boldsymbol{\tau}_i = \mathbf{m}_i \times \mathbf{B}_i \tag{5}$$

where $\mathbf{f}_i, \boldsymbol{\tau}_i \in \mathbb{R}^3$, ($i$ = 1,2,3), $\mathbf{m}_i$ is the magnetic dipole of the IPM located at position $\mathbf{P}_i$, and $\mathbf{B}_i$ is the magnetic field generated on the IPM at location $\mathbf{P}_i$ by the other IPMs and EPM. In the absence of a sufficiently strong magnetic field generated on the IPMs, the innate elasticity of crawler backbone and gravity will restore it to its original shape.

Expanding on equations (4) and (5), the forces $\mathbf{f}$ and torques $\boldsymbol{\tau}$ exerted on the IPMs due to the magnetic field generated by the EPM can be modelled as:

$$\mathbf{f}_{\mathrm{IPM}} = \frac{3\mu_0}{4\pi|\mathbf{r}_\alpha|^4}\left[\left(\hat{\mathbf{r}}_\alpha{}^{\mathrm{T}}\mathbf{m}_{\mathrm{IPM}}\right)\mathbf{m}_{\mathrm{EPM}} + \left(\hat{\mathbf{r}}_\alpha{}^{\mathrm{T}}\mathbf{m}_{\mathrm{EPM}}\right)\mathbf{m}_{\mathrm{IPM}}\right.$$

$$\left. + \left(\mathbf{m}_{\mathrm{EPM}}{}^{\mathrm{T}}\mathbf{m}_{\mathrm{IPM}} - \mathbf{5}\left(\hat{\mathbf{r}}_\alpha{}^{\mathrm{T}}\mathbf{m}_{\mathrm{EPM}}\right)\left(\hat{\mathbf{r}}_\alpha{}^{\mathrm{T}}\mathbf{m}_{\mathrm{IPM}}\right)\right)\hat{\mathbf{r}}_\alpha\right] \quad (6)$$

$$\boldsymbol{\tau}_{\mathrm{IPM}} = \mathbf{S}\{\mathbf{m}_{\mathrm{IPM}}\}\left[\frac{\mu_0}{4\pi|\mathbf{r}_\alpha|^3}\left(\mathbf{3}\hat{\mathbf{r}}_\alpha\hat{\mathbf{r}}_\alpha{}^{\mathrm{T}} - \mathbf{I}\right)\right]\mathbf{m}_{\mathrm{EPM}} \quad (7)$$

Where $\mathbf{r}_a$ is the distance between the EPM and the IPM, $\mathbf{m}_{\mathrm{EPM}}$ is the magnetic moment of the EPM, and $\mathbf{S}\{\mathbf{m}_{\mathrm{IPM}}\}$ is the skew-symmetric matrix packing of a vector used in the cross-product operation.

It is worth mentioning that given the size of the origami module, the IPMs were fixed and positioned such that the magnetic field generated between the two IPMs were not negligible. In particular, because the magnetic moment of the IPMs mirrored each other, the IPMs were constantly repelling each other and both IPMs experience an "outwards" rotational moment. In the absence of an externally generated magnetic field, the forces and torques acting on an IPM (IPMa) by the other (IPMb) can be modelled as:

$$\mathbf{f}_{\mathrm{IPMa}} = \frac{3\mu_0}{4\pi|\mathbf{r}_\beta|^4}\left[\left(\hat{\mathbf{r}}_\beta{}^{\mathrm{T}}\mathbf{m}_{\mathrm{IPMa}}\right)\mathbf{m}_{\mathrm{IPMb}} + \left(\hat{\mathbf{r}}_\beta{}^{\mathrm{T}}\mathbf{m}_{\mathrm{IPMb}}\right)\mathbf{m}_{\mathrm{IPMa}}\right.$$

$$\left. + \left(\mathbf{m}_{\mathrm{IPMb}}{}^{\mathrm{T}}\mathbf{m}_{\mathrm{IPMa}} - \mathbf{5}\left(\hat{\mathbf{r}}_\beta{}^{\mathrm{T}}\mathbf{m}_{\mathrm{IPMb}}\right)\left(\hat{\mathbf{r}}_\beta{}^{\mathrm{T}}\mathbf{m}_{\mathrm{IPMa}}\right)\right)\hat{\mathbf{r}}_\beta\right] \quad (8)$$

$$\boldsymbol{\tau}_{\mathrm{IPMa}} = \mathbf{S}\{\mathbf{m}_{\mathrm{IPMa}}\}\left[\frac{\mu_0}{4\pi|\mathbf{r}_\beta|^3}\left(\mathbf{3}\hat{\mathbf{r}}_\beta\hat{\mathbf{r}}_\beta{}^{\mathrm{T}} - \mathbf{I}\right)\right]\mathbf{m}_{\mathrm{IPMb}} \quad (9)$$

Where $\mathbf{r}_\beta$ is the distance between IPMa and IPMb, $\mathbf{m}_{\mathrm{IPMa}}$ and $\mathbf{m}_{\mathrm{IPMb}}$ are the magnetic moments of IPMa and IPMb respectively, and $\mathbf{S}\{\mathbf{m}_{\mathrm{IPMb}}\}$ is the skew-symmetric matrix packing of a vector used in the cross-product operation.

When the EPM was brought sufficiently close, the combination of the forces and torques acting on the IPM could be calculated as the summation of equations (6) and (8) for the total force, and equations (7) and (9) for the total torque.

Characterisation of the EPM. We measured the magnetic field generated by the north and south pole of the N52 EPM ($|\mathbf{B}|_{\mathrm{EPM}}$) at different distances |r| from its center using a magnetometer (TD8620, Tunkia Co., Ltd) as shown in Fig S5 (b). A linear motor rail (speed of 2000 pulse rev^{-1}) was used to vary the distance of the EPM from the sensor probe of the magnetometer. The initial and final position of the EPM relative to the sensor probe was measured, and the trajectory of the EPM and respective reading sensor reading of the magnetometer was videoed by an Iphone X at 30 FPS. The open-source Physlets Tracker video analysis and modelling tool (Copyright (c) 2019 Douglas Brown) [11] was used to track the relative trajectory of the EPM respective to the magnetometer's reading at each time step of 0.1 seconds. The absolute distance travelled by the center EPM in each time step was then calculated. I then best-fitted the results to obtain an estimation of the relationship between the magnetic field strength and the distance from the center of the magnet. As equation (3) implied an inverse cubic relationship between the magnetic field $\mathbf{B}$ generated and

the distance $|\mathbf{r}|$, I best-fitted the data accordingly in Origin (Version 2021b, OriginLab Corporation, Northampton, MA, USA). (Fig. S5 (c)-(d)).

From Origin, the relationship between the negative magnetic field $\mathbf{B}$ generated from the south pole as measured from the distance |r| is:

$$-\mathbf{B}\{\mathbf{r},\mathbf{m}\} = \frac{1.81 * 10^7}{4\pi|\mathbf{r}|^3} \tag{10}$$

While the relationship between the positive magnetic field $\mathbf{B}$ generated from the north pole as measured from the distance |r| is

$$\mathbf{B}\{\mathbf{r},\mathbf{m}\} = \frac{1.19 * 10^7}{4\pi|\mathbf{r}|^3} \tag{11}$$

Magnetic Actuation of the bistable V-fold. To estimate the strength of the magnetic field needed to deploy and un-deploy the bistable V-fold module, we anchored the origami mechanism onto a 3D printed base (PLA) and used a linear motor rail to vary the distance of the EPM from the center of the origami module (Fig S6). For analysis purposes, we set the center of the base of the origami as the origin and vary the y-position of the EPM with its x- and z- position fixed at zero relative to the origin (Fig 1 (c)-(d)). A mirror was used to minimize parallax error that may arise during recording. We then tracked the distance of the EPM relative from the origin (and by extension the origami mechanism), and the corresponding displacement d of the origami mechanism as a result of the folding and unfolding of the middle crease between the two degree-four vertices. We then calculated the corresponding estimated magnetic field of the EPM ($|\mathbf{B}|_{\mathrm{EPM}}$) from the best-fit equations obtained in Fig S6 (c)-(d). In total, three bistable V-fold samples were used and the distance of the EPM required to deploy and undeploy each sample were measured for 3 cycles (Fig S7). The distance from the center of the EPM to the bistable V-fold origami and the respective estimated magnetic field required to deploy and undeploy the samples were then averaged and the standard deviation (STDEV) was obtained from excel (Table. S1 and. S2).

We let $\mathbf{B}$ represent the non-uniform field generated by the EPM and abstract each IPM into a dipole with magnetic moment $\mathbf{m}$. When the IPM is placed in an applied magnetic field $\mathbf{B}$, the IPM is compelled to translate and rotate in an attempt to minimize magnetic energy [10]. In reality, the two effects of force and torque executes the actuation of the origami module simultaneously. However, for simplicity, we first consider the effects of force and torque separately.

We first explore the independent influences of magnetic torque $\boldsymbol{\tau}$ and $\mathbf{f}$ acting on the IPMs due to the applied magnetic field. In the case of a uniform magnetic field, the IPMs will only experience a magnetic torque $\boldsymbol{\tau}$ when a misalignment occurs between the magnetization moment $\mathbf{m}$ of the IPM and the direction of the magnetic field $\mathbf{B}$ [12]. In such a case, the imposed torque serves to rotate the IPMs such that its magnetic moment $\mathbf{m}$ will align with that of the magnetic field (Fig S8 (a)) in accordance with equation (5). In the case of our origami module, the torque directly results in a rotational moment $\mathbf{M}$ about the central crease of the origami module resulting in

reconfiguration, until it reaches the maximum structural limit is reached as shown in Fig S8 (b).

However, given that the magnetic field generated by the EPM is non-uniform, the IPM experiences a magnetic force $\mathbf{f}$ as well, causing the IPM to be translated in the direction as shown in Fig S9 to increase the magnetic energy, in accordance with the equation (4). As the IPMs are anchored to the origami mechanism, the imposed forces $\mathbf{f}$ act about a "pivot" point (green dots) due to the mechanical constraints of the origami mechanism, resulting in a rotational moment $\mathbf{M}$ about this pivot point.

Supplementary Note 4: Fabrication of the bistable origami crawler

Side Segments. The side segments of the origami crawler was fabricated from 50 µm thick PET sheet via the same laser cutting parameters as the bistable V-fold mechanism. For simplicity, we chose the same set of parameters for the side segments as that of the bistable V-fold (i.e. $\theta_{\mathrm{V}} = \theta_{\mathrm{H}} = 120°$, and $\theta_{\mathrm{B}} = 45°$). The number of units in the left and right segments were chosen to optimize the deformation of the crawler to achieve the desired shapes. When the number of units was too little, the origami crawler was not able to undergo sufficient deformation to achieve the required curvature without deploying. On the other hand, if the number of layers was too great, the origami crawler would be too underactuated and difficult to control.

Assembly of the origami crawler. Food-grade silicone adhesive (Cutorin 4672-B food grade silicone) was used to attach the IPMs and the segments together. Similar to the bistable origami V-fold mechanism, an N35 permanent disc magnet (5 mm diameter, 1 mm thickness, purchased from Titan™ Magnetics) was adhered to one external ends of the origami crawler in the desired orientation.

Supplementary Note 5: Magnetic actuation of the bistable origami crawler

Magnetic Actuation of the bistable V-fold. Besides the two IPMs located in the central segment, the other IPMs were positioned sufficiently far apart so that the effects they had on each other were negligible enough to be overcome by the inherent folding stiffness of the origami segments. To estimate the strength of the magnetic field needed to shape-morph the origami crawler and deploy and un-deploy the central bistable V-fold mechanism, we used a linear motor rail to vary the distance of the EPM from the center of the origami crawler (Fig S10). The origami crawler was actuated on Ecoflex 00-10 to reduce potential slippage that may arise from being actuated on a smoother surface with less friction. For analysis purposes, we set the center of the base of the origami crawler in its resting configuration as the origin and vary the y-position of the EPM with its x- and z- position fixed at zero relative to the origin. We then tracked the distance of the EPM relative from the origin (and by extension the origami crawler), and the corresponding displacement d of the left, center and right segments of the origami crawler. We then calculated the corresponding estimated magnetic field of the EPM ($|\mathbf{B}|_{\mathrm{EPM}}$) from the best-fit equations obtained in Fig S6 (c)-(d). The folding angle of the central unit of each segment (ϕ_{UL}, ϕ_{UC} and ϕ_{UR}) can be calculated via trigonometry using the cosine law as (Fig. S11):

$$\phi_{\mathrm{U}} = \cos^{-1}(1 - \frac{d^2}{2a^2}) \tag{12}$$

Directional locomotion. We used the same setup where a linear motor rail was used to vary the distance of the EPM from the base of the origami crawler (Fig. S12). The origami crawler was actuated on Ecoflex 00-10 to reduce potential slippage that may arise from being actuated on a smoother surface with less friction. For analysis purposes, we set the center of the base of the origami crawler in its resting configuration as the origin and vary the y-position of the EPM with its z- position fixed at zero relative to the origin. However, we fixed the x-position of the EPM at varying values relative to the central segment. We then tracked the y-position of the EPM relative to the origin (and by extension the origami crawler), and the corresponding x-position of the central unit of each segment was used to represent the displacement of the origami crawler. As per our nomenclature, the right segment indicated the front segment and a positive change in x position indicated forward displacement of the origami crawler.

Upon analysis of the mechanics of the locomotion of the bistable origami crawler and deduced that two factors mainly influence the ability of the bistable origami crawler to demonstrate directional locomotion: (i) ground reaction forces induced by magnetic forces (phases ①-②), and (ii) asymmetric coefficient of friction (phases ③-④). The two factors arose due to the shape-morphing of the origami crawler which generates frictional anisotropy, similar to that in annelids that modulate friction forces by changing their body shape, allowing them to pull themselves forward without the need for a complicated limb motion [13]. We consider the bistable crawler as two segments, front and back. In general, the friction force experienced by the front segment ($\mathbf{F}_{\mathrm{f1}}$) and back segment ($\mathbf{F}_{\mathrm{f2}}$) can be calculated as

$$\mathbf{F}_{\mathrm{f1}} = \mu_{\mathrm{f1}}\mathbf{N}_1 \quad (13)$$

$$\mathbf{F}_{\mathrm{f2}} = \mu_{\mathrm{f2}}\mathbf{N}_2 \quad (14)$$

Where μ_{f1} and μ_{f2} are the friction coefficients of the front segment and back segment respectively, $\mathbf{N_1}$ and $\mathbf{N_2}$ are the normal forces acting on the front segment and back segment respectively.

Phases ①-②

Due to the non-uniform magnetic field generated by the EPM on the front IPM, the front IPM experiences a greater amount of magnetic force as given by equation (4). The component of the magnetic force normal to the surface ($\mathbf{f}_{\mathrm{y}}$) results in the front end of the origami crawler exerting a greater amount of compressive force onto the surface, which results in a corresponding normal ground reaction force (GRF). The amount of friction force experienced by the front end ($\mathbf{F}_{\mathrm{f1}}$) and the back end ($\mathbf{F}_{\mathrm{f2}}$)can be approximated as follows:

$$\mathbf{F}_{\mathrm{f1}} = \mu_{\mathrm{f1}}\mathbf{N}_1 = \mu_{\mathrm{f1}}(m\mathbf{g} + \mathbf{F}_{\mathrm{GRF}}) \quad (15)$$

$$\mathbf{F}_{\mathrm{f2}} = \mu_{\mathrm{f2}}\mathbf{N}_2 = \mu_{\mathrm{f2}}(m\mathbf{g}) \quad (16)$$

where m is the mass of the segments, $\mathbf{g}$ is the gravitational vector, and $\mathbf{F}_{\mathrm{GRF}}$ is the ground reaction force. Since $\mathbf{F}_{\mathrm{GRF}} \geq 0$, $\mathbf{F}_{\mathrm{f1}} > \mathbf{F}_{\mathrm{f2}}$. Since the front-end experiences greater amount of friction, the front end serves as an anchor, while the back end slides forward (Fig S13 (a)). It is worth mentioning that a similar mode of friction modulation is also employed in caterpillars in real life, where they have been observed to exert different amounts of compressive forces on the substrate to induce different amounts of ground reaction forces, enabling them to develop tension within

its body that is then released to move segments forwards during the swing phase of a step [14]

Phases ③-④

Several works utilize feets exhibiting different friction coefficients on the front and rear end of the crawler such that alternating contact of the feets can enable translational motion [13], [15]. Similarly, the bistable origami crawler has anisotropic friction on the front end and the rear end, depending on which side of the ends are in contact with the surface. The side with the IPM exhibits lower friction coefficient, while the side with the PET fold exhibits higher friction coefficient. Due to the asymmetric inchworm shape the crawler adopts during phase ①-②, the IPM side of the front end of the crawler is in contact with the surface, while the PET side of the back end of the crawler is in contact with the surface. The amount of friction force experienced by the front end ($\mathbf{F}_{\mathrm{f1}}$) and the back end ($\mathbf{F}_{\mathrm{f2}}$)can be approximated as follows:

$$\mathbf{F}_{\mathrm{f1}} = \mu_{\mathrm{f1}}\mathbf{N}_1 = \mu_{f1}(m\mathbf{g}) \quad (17)$$

$$\mathbf{F}_{\mathrm{f2}} = \mu_{\mathrm{f2}}\mathbf{N}_2 = \mu_{f2}(m\mathbf{g}) \quad (18)$$

Since $\mu_{\mathrm{f2}} > \mu_{\mathrm{f1}}$, $\mathbf{F}_{\mathrm{f2}} > \mathbf{F}_{\mathrm{f1}}$. Since the back-end experiences greater amount of friction, the back end serves as an anchor, while the front end slides forward (Fig S13 (b)).

Position of the EPM respective to the bistable origami crawler

The x-position of the north pole of the EPM relative to the center of the origami crawler determines whether the origami crawler was able to achieve directional locomotion. As observed in Fig. S14 (a), positioning the north pole of the EPM ahead of the center of the origami crawler caused an initial forward displacement due to the anchoring of the front segment, pulling the rear end forward (①-②). When the EPM was not sufficiently ahead of the center of the origami crawler (6.5 mm in this case), the origami crawler would slide backwards after the initial forward displacement upon moving the EPM away, resulting in little to no net displacement (②-④). Hence, forward displacement cannot be achieved when the EPM is not sufficiently positioned ahead of the origami crawler, as this will lead to less asymmetric deformation of the origami crawler as observed in phase ②, and $\mu_{\mathrm{f2}} \leq \mu_{\mathrm{f1}}$, resulting in either the front end acting as an anchor (or neither ends acting as an anchor), and hence the back end slides backwards instead of the front end sliding forward.

Conversely, when the EPM was placed too far ahead of the center of the origami crawler (18 mm in this case) as in Fig. S14 (b), forward displacement can be observed. However, as the magnitude of the component of the magnetic force that is perpendicular to the travelling surface ($\mathbf{f}_x$) is significantly large enough to overcome the frictional force, forward slippage will occur, which can lead to uncontrollable displacements which is hence undesirable.

Crawling speed of the origami crawler. We manually actuated the origami crawler on Ecoflex 00-10 at different frequencies for ~ 20 s. We tracked the position of the center of the crawler over time using the Physlets tracker, which was used to reflect the distance the crawler travelled (Fig. S15 (a)). We chose to use Ecoflex as the travelling material as it was commonly used as tissue phantoms and models in

literature as they possessed similar stiffness to that of biological tissues [16], [17], [18], [19], [20], [21]. We actuated at origami crawler at three different frequencies for three cycles each: 1.0 Hz, 1.25 Hz and 1.5 Hz. A metronome was used to ensure actuation of the origami crawler at a consistent frequency (60 bpm = 1.0 Hz, 75 bpm = 1.25 Hz, 90 bpm = 1.5 Hz). The average displacement and standard deviation travelled by the origami crawler was then taken by averaging the displacement values at each time stamp was obtained from excel. From Fig. S15 (d), we observed that the first cycle (C1) of the actuation of the origami crawler at 1.5 Hz is drastically different from the other two cycles and hence excluded from analysis. The travelling speed of the origami crawler is calculated by dividing the total distance travelled by the origami crawler by the total time (~20 s) required for it to cover that distance. The normalized travelling speed (bodylength/s) of the origami crawler is calculated as follows:

$$\text{bodylength s}^{-1} = \frac{\text{average speed (mm s}^{-1})}{\text{origami crawler length (mm)}} = \frac{\text{average speed (mm s}^{-1})}{22 \text{ mm}} \qquad (19)$$

The travelling speed, normalized travelling speed and the respective standard deviation of the origami crawler at each actuation frequency is calculated from excel as shown in Table. S3.

Tilting of the origami crawler. We used the same setup where a linear motor rail was used to vary the distance of the EPM from the base of the origami crawler (Fig. S16). The origami crawler was actuated on Ecoflex 00-10 (~ 0.5 mm thick) to reduce potential slippage that may arise from being actuated on a smoother surface with less friction. For analysis purposes, we set the center of the base of the origami crawler in its resting configuration as the origin and vary the z-position of the EPM with its x-position fixed at zero relative to the origin. However, we fixed the y-position of the EPM at 25 mm. We then tracked the z-position of the EPM relative to the origin (and by extension the origami crawler), and the corresponding z-position of the origami crawler was used to calculate the tilt angle of the origami crawler.

Navigation of the origami crawler on porcine small intestine. We obtained porcine small intestine from food2homes. A segment of the small intestine was cut open and laid flat to expose the intestinal wall on a 3D printed phantom and adhered using cyanoacrylate to prevent slipping of the small intestine during locomotion of the origami crawler. Most of the mucin was removed from the small intestine surface.

Supplementary Note 6: Microneedles delivery and insertion

Verification of microneedles insertion. The topology of the microneedles of the CASMA patch as viewed under an optical microscope (Binocular 50000, Tikuo Inc., China) is shown in Fig. S17 (a), while the topology of an un-punctured Ecoflex (~ 0.5 mm thick) is shown in Fig. S17 (b). From Fig. S17 (c), visible puncture marks can be observed in the Ecoflex film from the microneedles delivered by the origami crawler. To verify that the puncture marks observed is attributed to the insertion of microneedles, we compared the puncture marks created by the insertion of microneedles by the origami crawler with that made by a commercial spring-loaded applicator (MPatch Mini Applicator, purchased from Micropoints Technology Pte Ltd), which was often used for epidermal microneedle insertion (Fig. S17 (d)). From Fig. S17 (d), we observed an array of puncture holes that verifies that the marks observed on the Ecoflex are due to the insertion of microneedles. The marks observed in both Fig. S17 (c) and (d) bear resemblance, verifying successful puncture of the microneedles into the Ecoflex film by the origami crawler.

It is worth noting that in contrast to the puncture marks made by the commercial spring-loaded applicator, the puncture holes made by the origami spring were less distinct. We attributed this to two reasons. Firstly, while the spring applicator was used to insert the microneedles on a flat uniform surface, the origami spring was used for endoluminal microneedles insertion, which resulted in non-uniform force distribution due to the insertion of microneedles on a curved surface in a tubular environment. Secondly, the amount of force that the origami spring was able to generate from the snap-through bistability was likely to be lesser than that of the spring applicator, and hence the microneedles deployed by the origami spring may not have penetrated the Ecoflex film as deeply as those deployed by the commercial spring applicator. We believe that the force-loading capacity of the origami spring can be further enhanced to improve its microneedles insertion capability. In addition, we believe that coupled with the peristaltic motion in the small intestine, sufficient compressive forces can be exerted by the origami crawler on the intestinal wall for greater contact and penetration insertion of the microneedles for biomedical applications [22].

Supplementary Figures

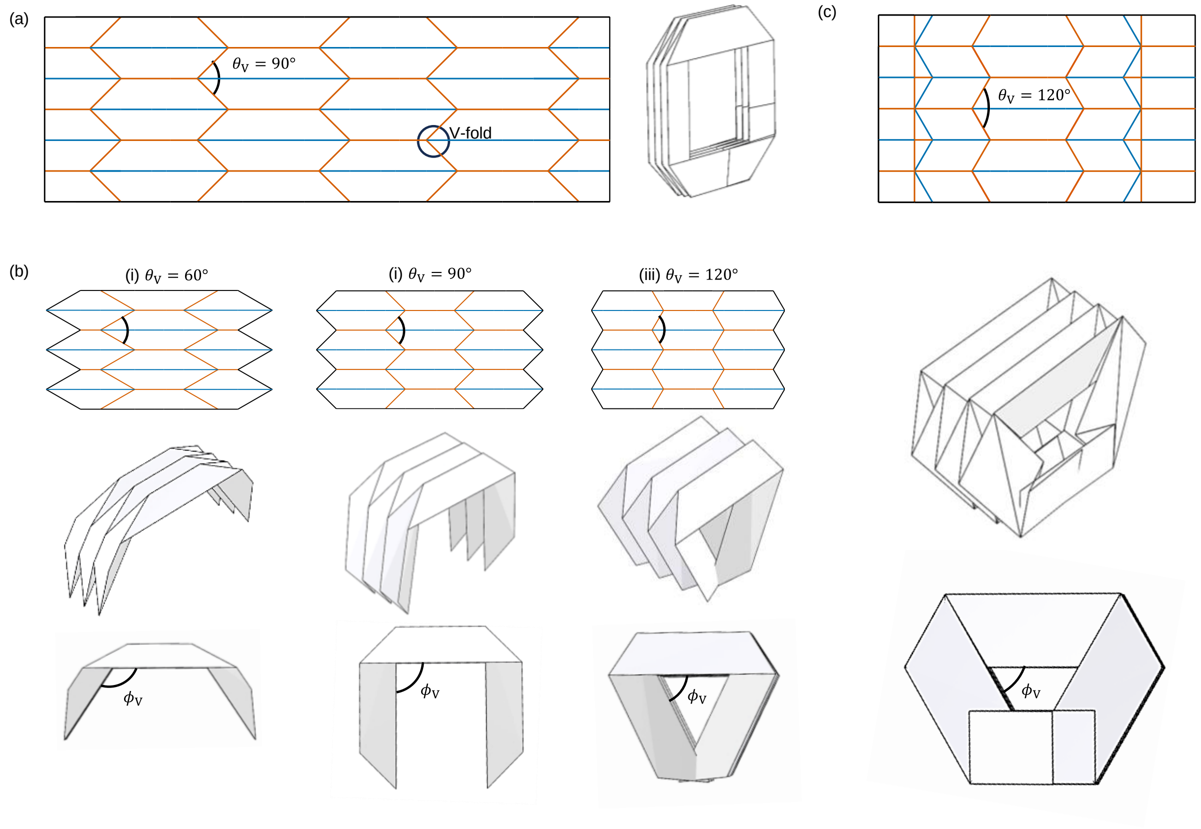

Figure S1 Influence of V-fold angle (θ_V). (a) Crease pattern and folded counterpart of the accordion bellows consisting of alternating V-folds with an angle $\boldsymbol{\theta_V}$ of 90°. The circle indicates the distinctive V shape where the three mountain folds (vermillion lines) and one valley fold (blue) meet at a node. (b) The crease patterns and the corresponding simulated origami structures when $\boldsymbol{\theta_V}$ = (i) 60°, (ii) 90° and (iii) 120°. A larger folding angle ($\boldsymbol{\theta_V}$) results in a smaller closing angle ($\boldsymbol{\phi_V}$) of the structure and vice-versa. (c) The modified origami crease pattern allowing for folding of V-fold origami with larger folding angles. Here, the folding angle $\boldsymbol{\theta_V}$ = 120°.

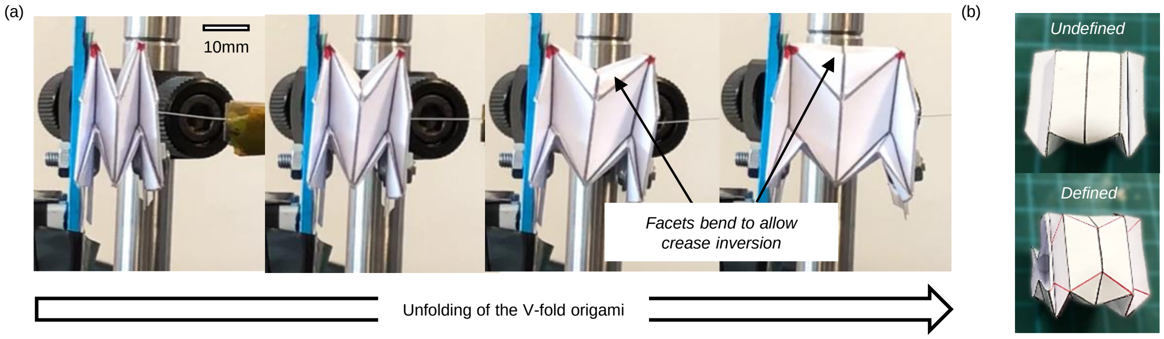

Figure S2 Influence of Hidden angle (θ_{H}). (a) Modified bistable V-fold origami demonstrated bistability as a result of energy-scale dependent DoF due to its non-rigid foldable nature. Unfolding the V-fold origami beyond a certain point caused the facets to bend, enabling crease inversion. At this point, the origami entered a different stable configuration. (b) The folded origami with undefined (top) and defined bending creases (bottom).

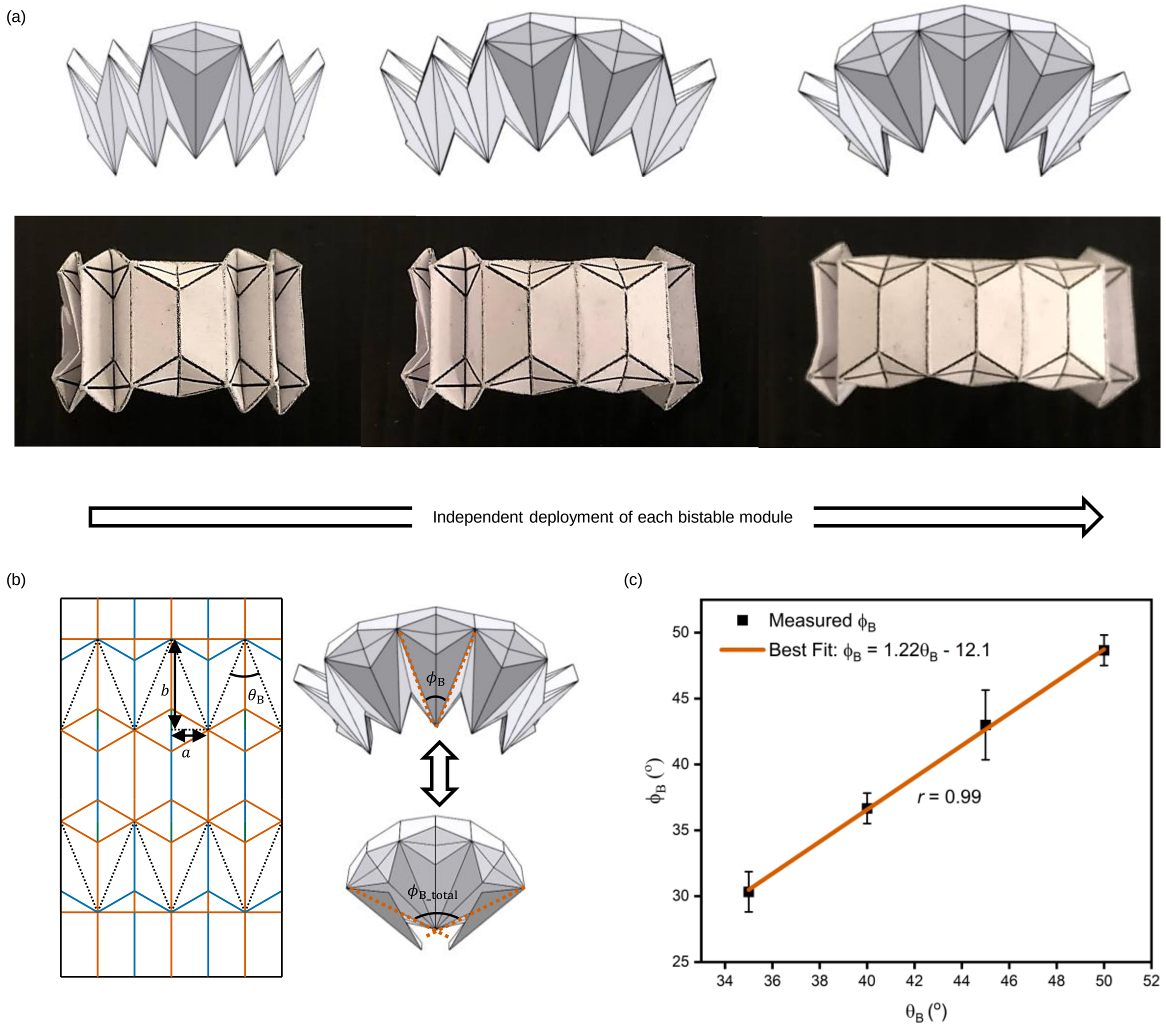

Figure S3 Influence of Bending angle (θ_B). (a) Combining several bistable V-fold modules result in an aggregated structure where each module can deploy independently of its neighbouring modules. (b) Bending radius $\boldsymbol{\Phi}_{\mathbf{B}}$ of each deployed V-fold origami module was dependent on the angle $\boldsymbol{\theta}_{\mathbf{B}}$, which can be calculated given the height $\boldsymbol{b}$ and half-length $\boldsymbol{a}$ of the triangulated V-fold in each unit of the bistable V-origami crease pattern. The minimum bending angle of a fully deployed origami structure consisting of aggregated bistable V-fold origami modules was given as $\boldsymbol{\phi}_{\mathbf{B\_total}}$. (c) Linear relationship between $\boldsymbol{\Phi}_{\mathbf{B}}$ and $\boldsymbol{\theta}_{\mathbf{B}}$ for the average of three samples. The standard error bars are calculated using standard deviation in excel.

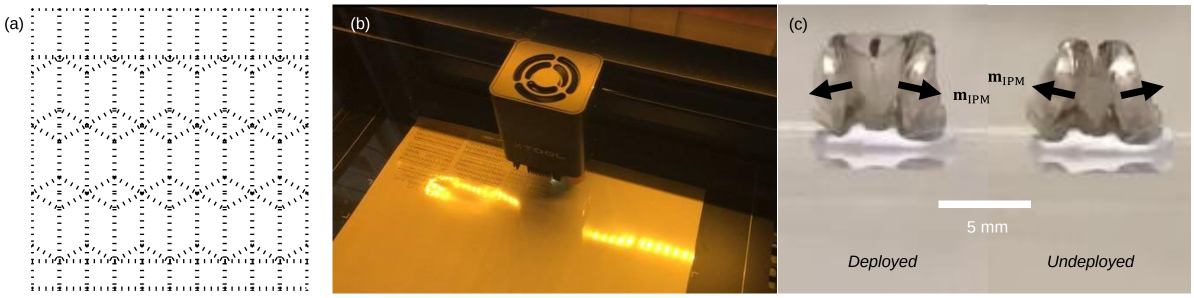

Figure S4 Fabrication of miniature bistable V-fold module. (a) Crease pattern of the origami V-fold where the crease lines are drawn as perforated holes. (b) Laser cutting of crease pattern on 50 μm thick PET sheets. (c) Integration of 5 mm x 1 mm discs magnets into the bistable V-fold origami module. IPMs were tucked into the ends of the origami module and oriented in a way that that their magnetic moments ($\mathbf{m}_{\mathrm{IPM}}$) mirrored each other.

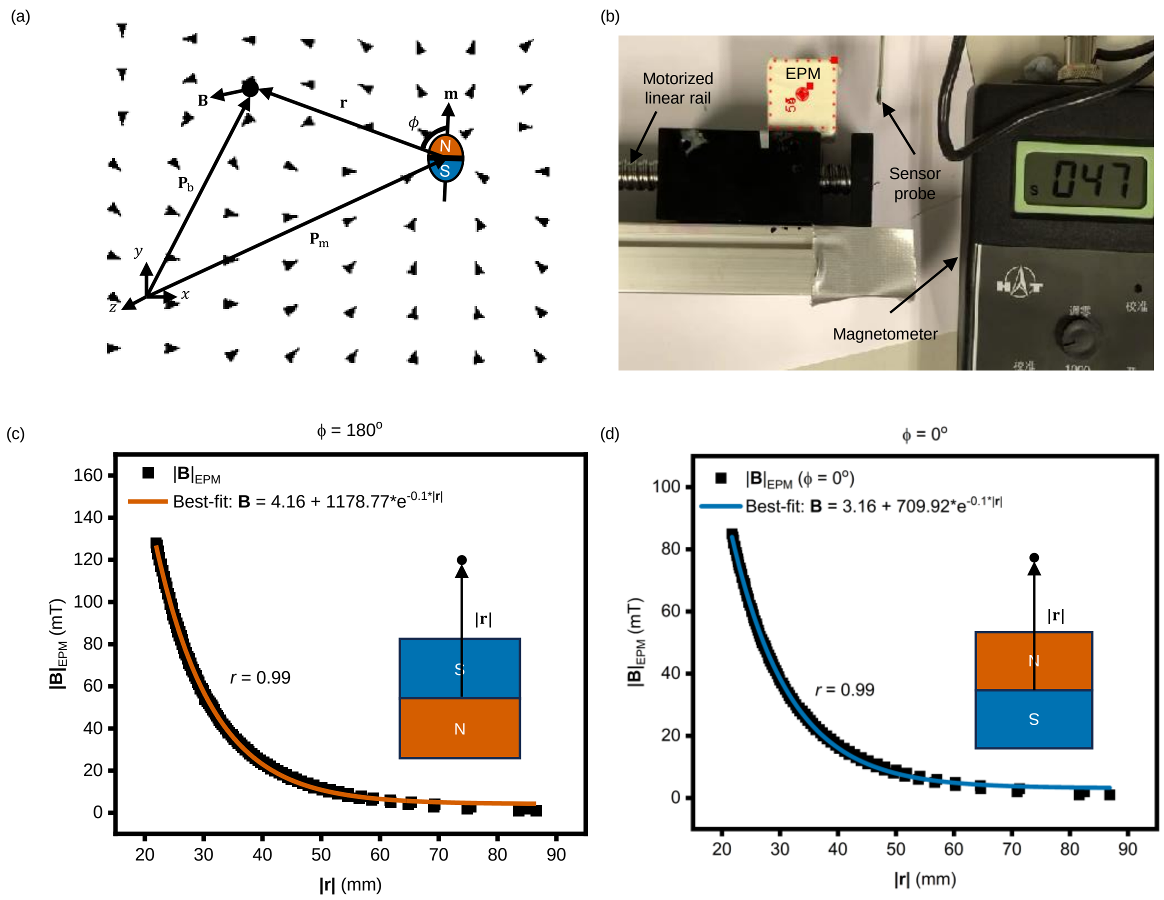

Figure S5 Characterisation of EPM. (a) Representation of magnetic field (**B**) measured at point $\mathbf{P_b}$ by the external permanent magnet located at $\mathbf{P_m}$ and oriented at angle $\boldsymbol{\phi}$ as abstracted as a magnetic dipole with magnetic moment $\mathbf{m}$. $\mathbf{r}$ is the distance between the position of measurement $\mathbf{P_b}$ and the position of the external magnet $\mathbf{P_m}$. (b) Experimental setup where a linearised motor was used to vary the distance between the EPM and the magnetometer. The relative trajectory of the EPM (red circle) and respective magnetic field measurement reflected on the magnetometer was tracked using the open-source Physlets Tracker video analysis and modelling tool (c) Characterisation of the magnitude of the magnetic field generated by the EPM ($|\mathbf{B}|_{\mathbf{EPM}}$) at different distances $|\mathbf{r}|$ as measured from its south pole and (d) north pole. The data is best-fitted in origin using an inverse cubic relationship.

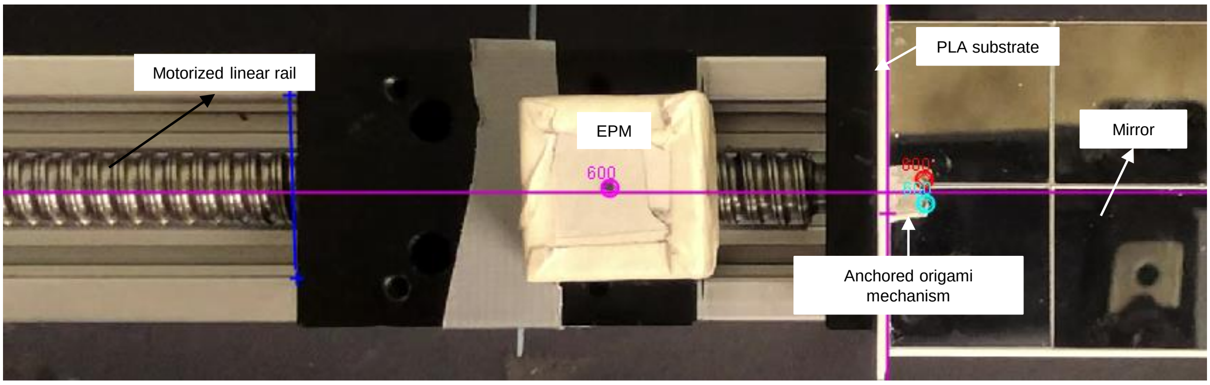

Figure S6 Experimental setup for the magnetic Actuation of bistable V-fold origami mechanism. A motorized linear rail was used to vary the y-position of the EPM relative from the origami mechanism to modulate the strength of the induced magnetic field throughout the actuation of the mechanism.

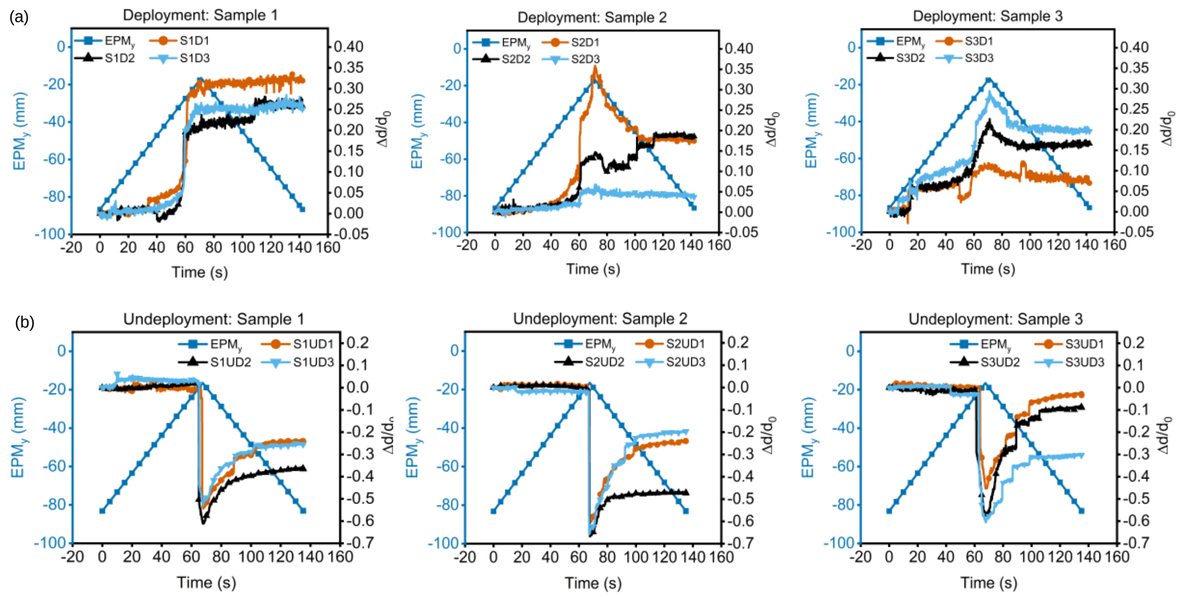

Figure S7 Distance from the center of the EPM to the bistable V-fold origami vs deformation of the origami mechanism. (a) Deployment of samples 1 to 3, each for 3 cycles. (b) Undeployment of samples 1 to 3, each for 3 cycles.

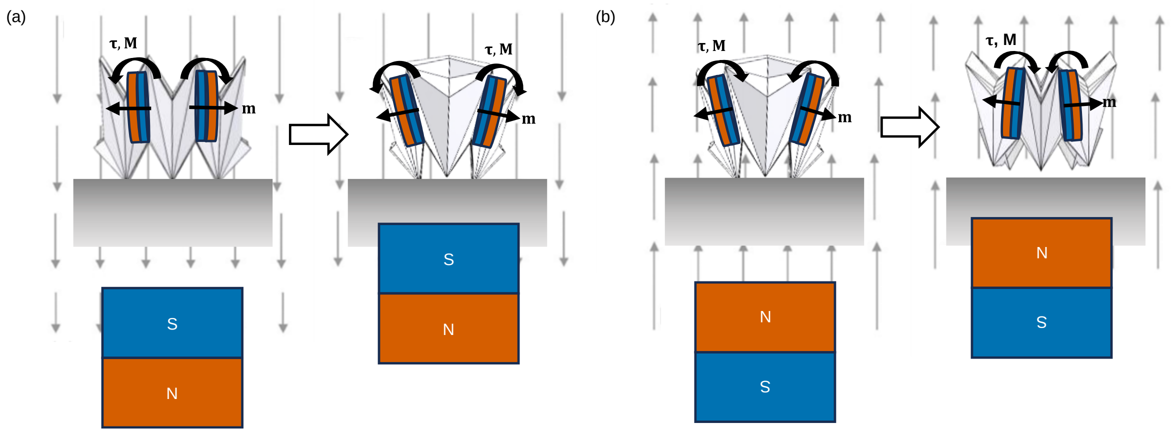

Figure S8 Effect of magnetic torque. Magnetic torque $\boldsymbol{\tau}$ causes rotational moment $\mathbf{M}$ of IPMs with magnetic moment $\mathbf{m}$ leading to the deformation and (a) deployment of the origami module, and (b) undeployment of the origami module.

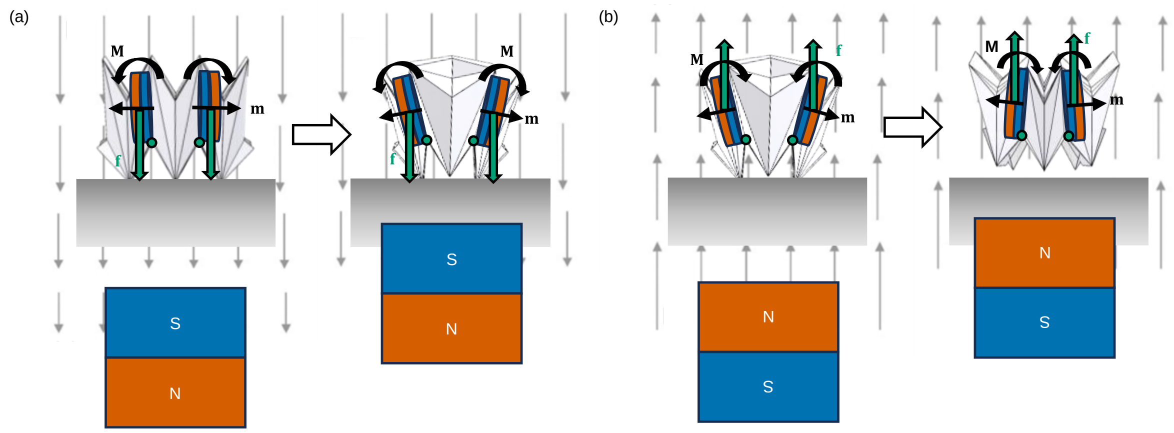

Figure S9 Effect of magnetic force. Magnetic force $\mathbf{f}$ causes rotational moment $\mathbf{M}$ of IPMs with magnetic moment $\mathbf{m}$ leading to the deformation and (a) deployment of the origami module, and (b) undeployment of the origami module.

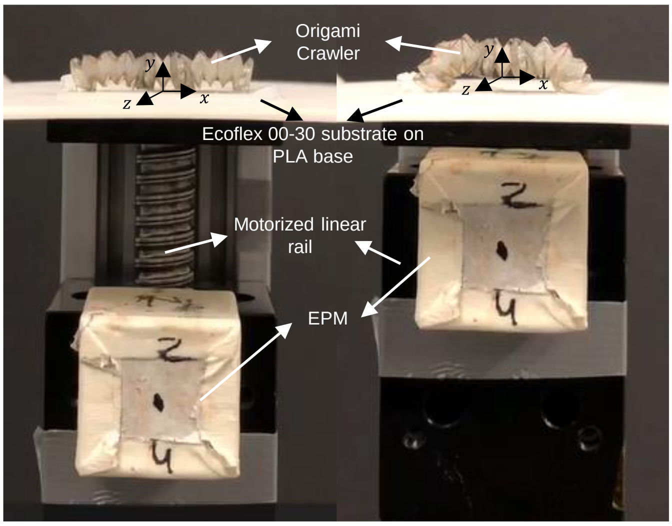

Figure S10 Experimental setup for the magnetic actuation of the origami crawler to induce shape-morphing between the deployed and undeployed state. A motorized linear rail was used to vary the y-position of the EPM relative from the origami crawler to modulate the strength of the induced magnetic field throughout the actuation of the mechanism.

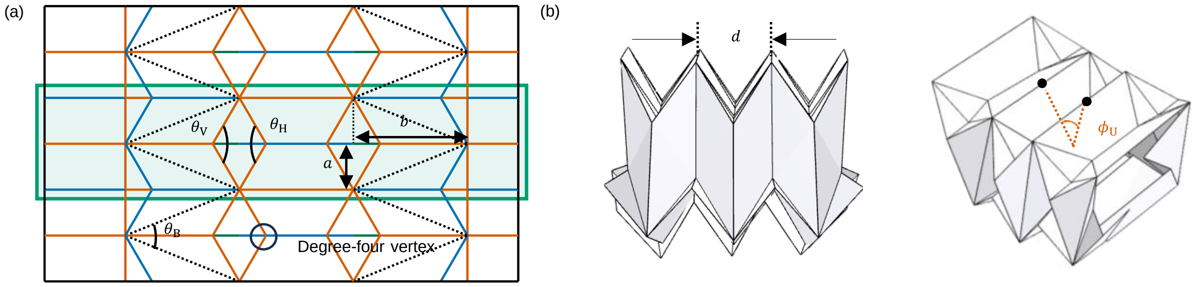

Figure S11 Characterisation of Folding angle ($\boldsymbol{\phi_U}$). The folding angle $\boldsymbol{\phi_U}$ of the origami V-fold can be calculated via trigonometry. $\boldsymbol{\theta_V}$, $\boldsymbol{\theta_H}$, $\boldsymbol{\theta_B}$, $\boldsymbol{a}$ and $\boldsymbol{b}$ refers to the V-fold angle, the hidden angle, the bending angle, the half-length and height of the triangulated V-fold in each unit of the bistable V-origami crease pattern respectively. $\boldsymbol{d}$ is the displacement the origami module undergoes under linear folding and unfolding.

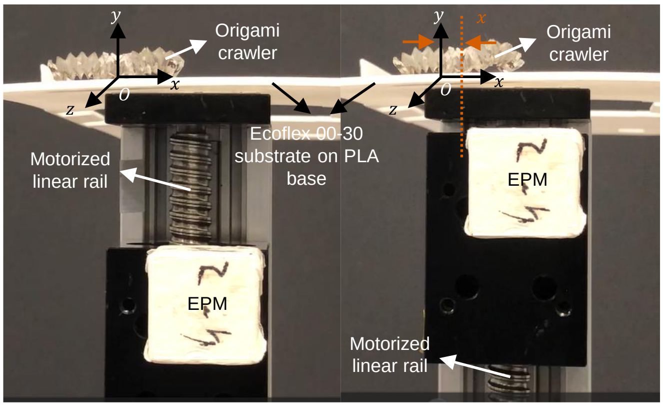

Figure S12 Experimental set for inducing directional locomotion of origami crawler. A motorized linear rail was used to vary the $\boldsymbol{y}$-position of the north pole of the EPM relative to the origami crawler. The $\boldsymbol{z}$-position of the EPM relative to the origami crawler was fixed at zero, while the $\boldsymbol{x}$-position of the EPM relative to the origami crawler was fixed at a fix distance away from the origin. The $\boldsymbol{x}$-position of the central segment of the origami crawler was tracked as a representation of the displacement of the crawler.

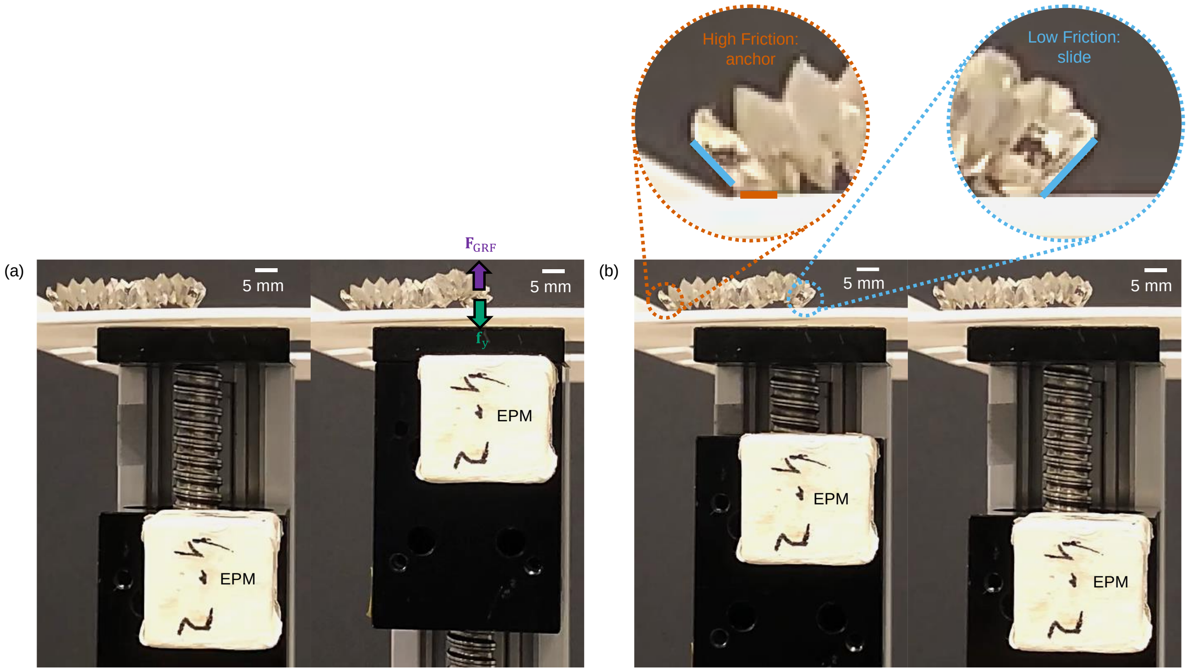

Figure S13 Asymmetrical shape deformation modulates friction anisotropy for directional locomotion. (a) In phases ①-②, the front end of the origami crawler experiences ground reaction forces and hence larger friction forces, acting as the anchor while the back end slides forward. $\mathbf{F_{GRF}}$ and $\mathbf{f}_y$ represents the normal ground reaction force and the component of the magnetic force normal to the surface respectively. (b) In phases ③-④, as the shape asymmetry results in different contact surfaces of the origami crawler, the back end has higher friction coefficient, acting as the anchor while the front end slides forward.

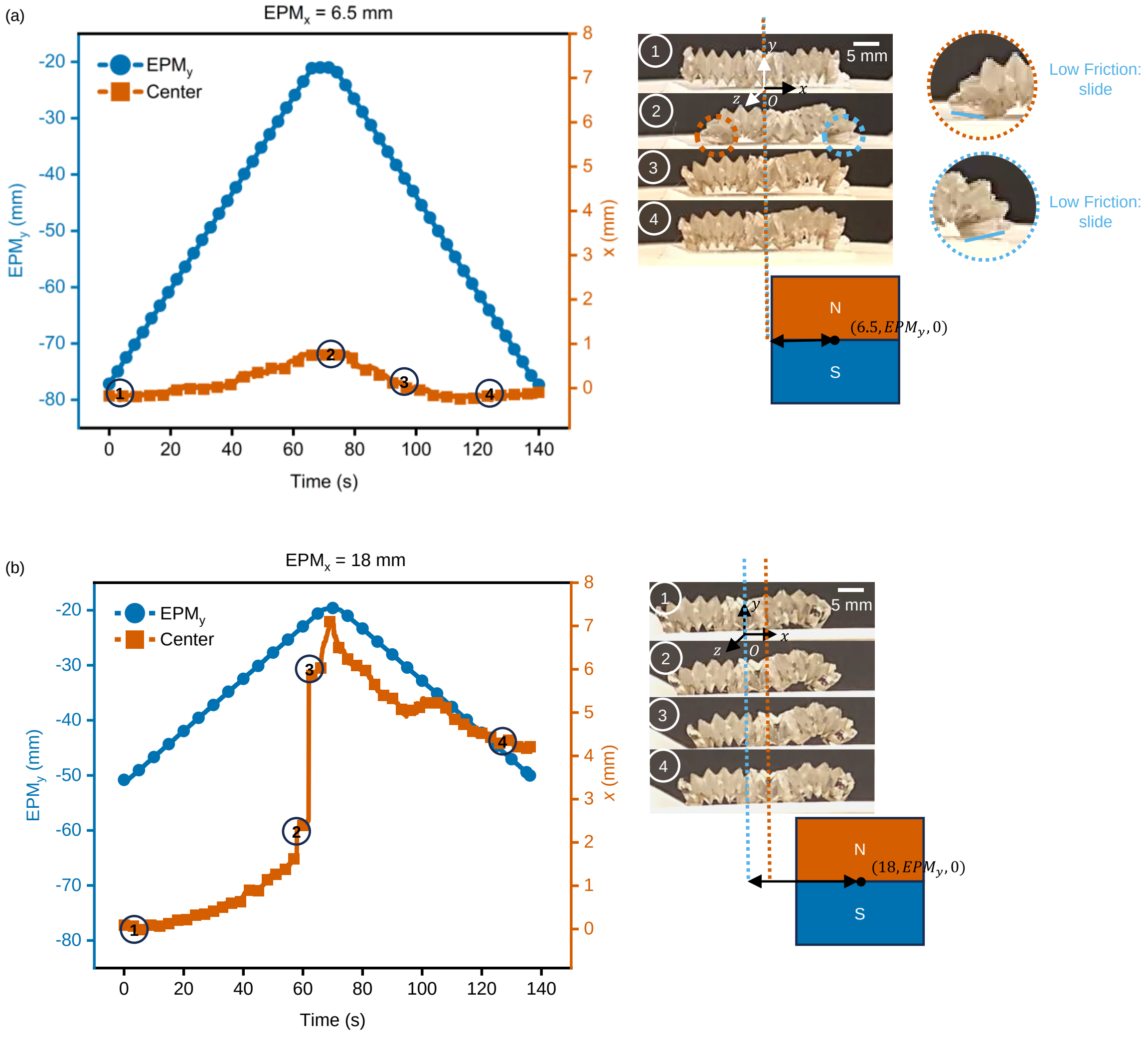

Figure S14 Position of the EPM relative to the front end of the origami crawler affects directional locomotion. (a) Placing the north pole of the EPM too close to the center of the origami crawler ($\boldsymbol{EPM_x} = \mathbf{6.5\ mm}$) resulted in little to no net displacement. (b) Placing the north pole of the EPM too far ahead from the center of the origami crawler ($\boldsymbol{EPM_x} = \mathbf{18\ mm}$) resulted in undesirable slippage.

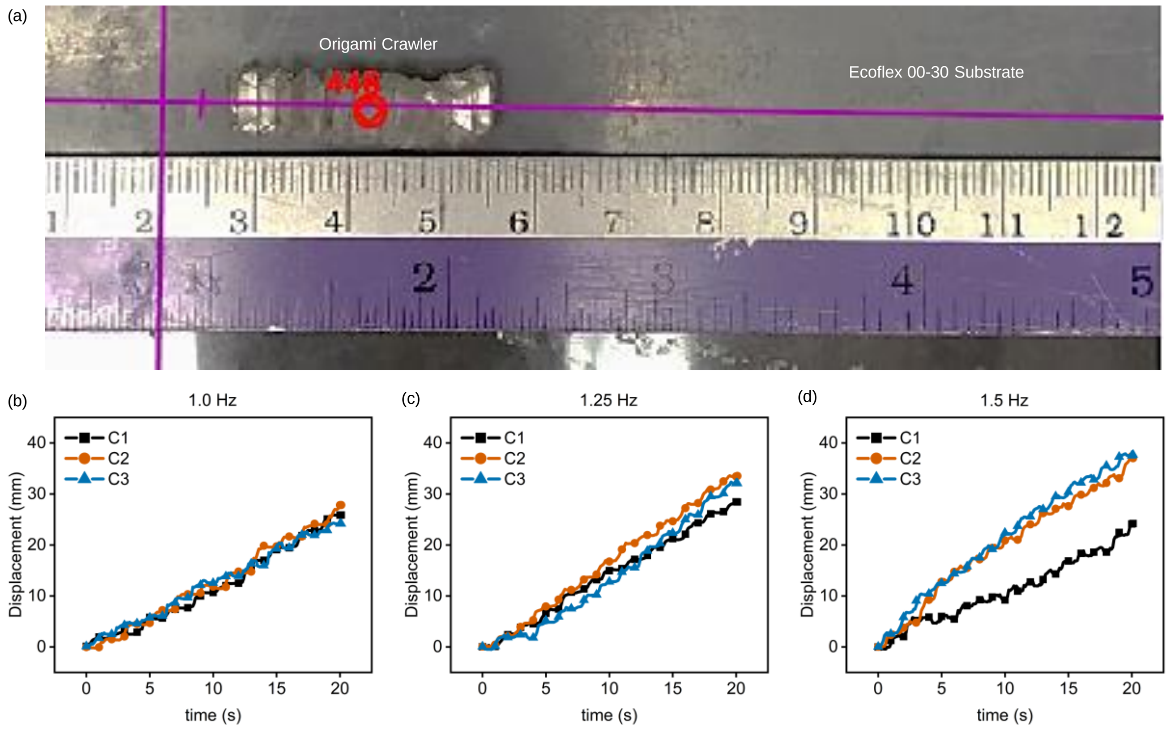

Figure S15 Speed of the Origami crawler. (a) The origami crawler was manually actuated on Ecoflex 00-10 at different frequencies. The position of the center of the crawler over time using the Physlets tracker, which was used to reflect the distance the crawler travelled for actuation frequencies of (b) 1.0 Hz, (c) 1.25 Hz, and (c) 1.5 Hz.

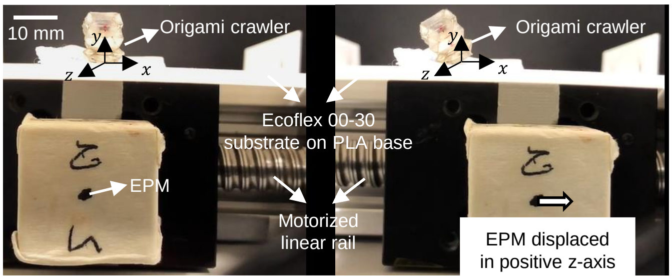

Figure S16 Experimental setup for the tilting of origami crawler. A motorized linear rail was used to vary the $\boldsymbol{z}$-position of the north pole of the EPM relative to the origami crawler. The $\boldsymbol{x}$-position of the EPM relative to the origami crawler was fixed at zero, while the $\boldsymbol{y}$-position of the EPM relative to the origami crawler was fixed at a fix distance away from the origin (25 mm). The tilting angle of the central segment of the origami crawler was tracked.

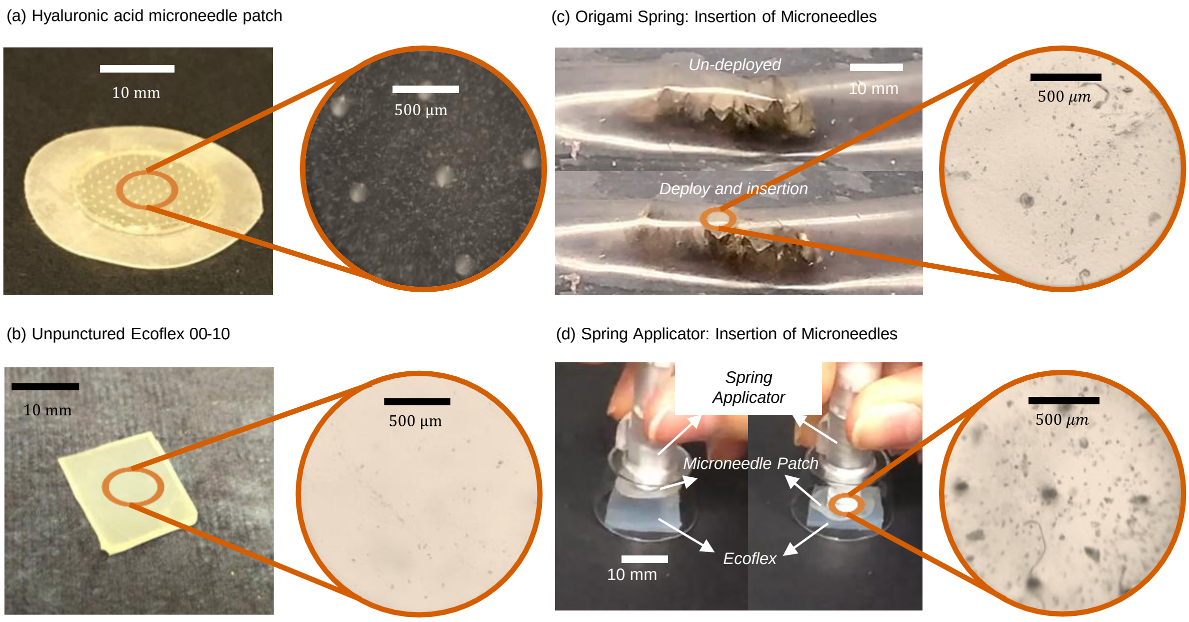

Figure S 17 Optical microscope images for the verification of the insertion of microneedles into an Ecoflex film. (a) Topology of the CASMA microneedles patch used. (b) Topology of an un-punctured Ecoflex film. (c) Ecoflex film punctured by microneedles inserted by the origami crawler. (d) Ecoflex film punctured by microneedles inserted by a commercial spring-loaded applicator.

Supplementary Tables

Table. S1 Magnetic field strength required to deploy the bistable V-fold mechanism

Sample	Cycle	$\mathrm{EPM_y}$ (mm)	STDEV	B-field (mT)	STDEV
S1	S1D1	26.5		77.7	
	S1D2	28.7		60.7	
	S1D3	28.1		64.7	
S2	S2D1	26.4		78.6	
	S2D2	26.5		77.7	
	S2D3	27.2		72.0	
S3	S3D1	28.5		62.0	
	S3D2	26.8		75.2	
	S3D3	26.1		81.3	
Average		27.2	1.02	72.2	7.80

Table. S2 Magnetic field strength required to undeploy the bistable V-fold mechanism

Sample	Cycle	EPM_y (mm)	STDEV	B-field (mT)	STDEV
S1	S1UD1	16.9	[redacted]	196.4	[redacted]
	S1UD2	19.1		136.7	
	S1UD3	18.9		141.0	
S2	S2UD1	17.1		189.7	
	S2UD2	16.6		207.1	
	S2UD3	16.9		196.4	
S3	S3UD1	20.1		115.8	
	S3UD2	22.7		80.8	
	S3UD3	20.8		104.6	
Average		18.8	2.13	152.0	46.6

Table. S3 Travelling speed of the origami crawler at different actuation frequencies

Actuation freq	Cycle	Speed (mm/s)	Bodylength s^{-1}	Average Speed (mm/s)	Speed STDEV	Average Bodylength s^{-1}	Bodylength s^{-1} STDEV
1.0 Hz	C1	1.28	0.0583	1.29	0.0958	0.0585	0.00436
	C2	1.39	0.0630				
	C3	1.19	0.0543				
1.25 Hz	C1	1.41	0.0642	1.56	0.0129	0.0708	0.00587
	C2	1.66	0.0756				
	C3	1.59	0.0724				
1.5 Hz	C2	1.84	0.0836	1.86	0.0234	0.0844	0.00106
	C3	1.87	0.0851				

Legends for Supplementary Movies

Supplementary Movie S1. Deployment and undeployment of bistable V-fold

Supplementary Movie S2. Steering on porcine tissue

Supplementary Movie S3. Crawling in confined spaces

Supplementary Movie S4. Crawling in porcine small intestine

Supplementary Movie S5. Microneedle delivery and insertion

Supplementary Data

Raw Microscopy Images (JPG files)

Source files of the collected data (Unicode Origin Graph)

Supplementary References

[1] P. Jackson, *Folding Techniques for Designers-From Sheet to Form*. Laurence King Publishing, 2011.

[2] S. Miyashita, C. D. Onal, and D. Rus, "Self-pop-up cylindrical structure by global heating," in *2013 IEEE/RSJ International Conference on Intelligent Robots and Systems*, IEEE, 2013, pp. 4065–4071.

[3] A. Ghassaei, E. D. Demaine, and N. Gershenfeld, "Fast, interactive origami simulation using GPU computation," *Origami*, vol. 7, pp. 1151–1166, 2018.

[4] A. Reid, F. Lechenault, S. Rica, and M. Adda-Bedia, "Geometry and design of origami bellows with tunable response," *Phys Rev E*, vol. 95, no. 1, p. 13002, 2017.

[5] R. Connelly, I. Sabitov, and A. Walz, "The bellows conjecture," *Beitr. Algebra Geom*, vol. 38, no. 1, pp. 1–10, 1997.

[6] J. Butler *et al.*, "Highly compressible origami bellows for harsh environments," in *International Design Engineering Technical Conferences and Computers and Information in Engineering Conference*, American Society of Mechanical Engineers, 2016, p. V05BT07A001.

[7] J. L. Silverberg *et al.*, "Origami structures with a critical transition to bistability arising from hidden degrees of freedom," *Nat Mater*, vol. 14, no. 4, pp. 389–393, 2015, doi: 10.1038/nmat4232.

[8] D. Melancon, A. E. Forte, L. M. Kamp, B. Gorissen, and K. Bertoldi, "Inflatable origami: Multimodal deformation via multistability," *Adv Funct Mater*, vol. 32, no. 35, p. 2201891, 2022.

[9] V. K. Venkiteswaran, D. K. Tan, and S. Misra, "Tandem actuation of legged locomotion and grasping manipulation in soft robots using magnetic fields," *Extreme Mech Lett*, vol. 41, p. 101023, 2020.

[10] J. J. Abbott, E. Diller, and A. J. Petruska, "Magnetic Methods in Robotics," *Annu Rev Control Robot Auton Syst*, vol. 3, no. 1, pp. 57–90, May 2020, doi: 10.1146/annurev-control-081219-082713.

[11] D. Brown and A. J. Cox, "Innovative uses of video analysis," *Phys Teach*, vol. 47, no. 3, pp. 145–150, 2009.

[12] Z. Yang and L. Zhang, "Magnetic Actuation Systems for Miniature Robots: A Review," *Advanced Intelligent Systems*, vol. 2, no. 9, p. 2000082, 2020, doi: 10.1002/aisy.202000082.

[13] C. D. Onal, R. J. Wood, and D. Rus, "An origami-inspired approach to worm robots," *IEEE/ASME Transactions on Mechatronics*, vol. 18, no. 2, pp. 430–438, 2013, doi: 10.1109/TMECH.2012.2210239.

[14] M. A. Simon, S. J. Fusillo, K. Colman, and B. A. Trimmer, "Motor patterns associated with crawling in a soft-bodied arthropod," *Journal of Experimental Biology*, vol. 213, no. 13, pp. 2303–2309, 2010, doi: 10.1242/jeb.039206.

[15] Z. Qiji *et al.*, "Soft robotic origami crawler," *Sci Adv*, vol. 8, no. 13, p. eabm7834, Apr. 2022, doi: 10.1126/sciadv.abm7834.

[16] S. Miyashita, S. Guitron, K. Yoshida, S. Li, D. D. Damian, and D. Rus, "Ingestible, controllable, and degradable origami robot for patching stomach wounds," *Proc IEEE*

Int Conf Robot Autom, vol. 2016-June, no. 5, pp. 909–916, 2016, doi: 10.1109/ICRA.2016.7487222.

[17] J. L. Sparks *et al.*, "Use of silicone materials to simulate tissue biomechanics as related to deep tissue injury," *Adv Skin Wound Care*, vol. 28, no. 2, pp. 59–68, 2015.

[18] M. Engers, K. W. Stewart, J. Liu, and P. P. Pott, "Development of a realistic venepuncture phantom," *Current Directions in Biomedical Engineering*, vol. 6, no. 3, pp. 402–405, 2020.

[19] S. Miyashita, S. Guitron, K. Yoshida, S. Li, D. D. Damian, and D. Rus, "Ingestible, controllable, and degradable origami robot for patching stomach wounds," *Proc IEEE Int Conf Robot Autom*, vol. 2016-June, no. 5, pp. 909–916, 2016, doi: 10.1109/ICRA.2016.7487222.

[20] J. L. Sparks *et al.*, "Use of silicone materials to simulate tissue biomechanics as related to deep tissue injury," *Adv Skin Wound Care*, vol. 28, no. 2, pp. 59–68, 2015.

[21] M. Engers, K. W. Stewart, J. Liu, and P. P. Pott, "Development of a realistic venepuncture phantom," *Current Directions in Biomedical Engineering*, vol. 6, no. 3, pp. 402–405, 2020.

[22] G. Traverso *et al.*, "Microneedles for drug delivery via the gastrointestinal tract," *J Pharm Sci*, vol. 104, no. 2, pp. 362–367, 2015, doi: 10.1002/jps.24182.
