# Supplementary material for: Untethered bistable origami crawler for confined applications
Source: Commun Eng. 2024 Oct 30;3:150. doi: 10.1038/s44172-024-00294-1 (PMC11525557; doi:10.1038/s44172-024-00294-1)
Supplement: Supplementary file 3 — Description of Additional Supplementary Files [file 44172_2024_294_MOESM3_ESM.pdf]

Description of Additional Supplementary Files

File name: Supplementary Movie 1

Description: Deployment and undeployment of bistable V-fold

File name: Supplementary Movie 2

Description: Steering on porcine tissue

File name: Supplementary Movie 3

Description: Crawling in confined spaces

File name: Supplementary Movie 4

Description: Crawling in porcine small intestine

File name: Supplementary Movie 5

Description: Microneedle delivery and insertion

File name: Supplementary Data 1

Description: zip file containing origin files and data used to create the main and supplementary figures
