# Supplementary figures and images for: Untethered bistable origami crawler for confined applications
Source: Commun Eng. 2024 Oct 30;3:150. doi: 10.1038/s44172-024-00294-1 (PMC11525557; doi:10.1038/s44172-024-00294-1)

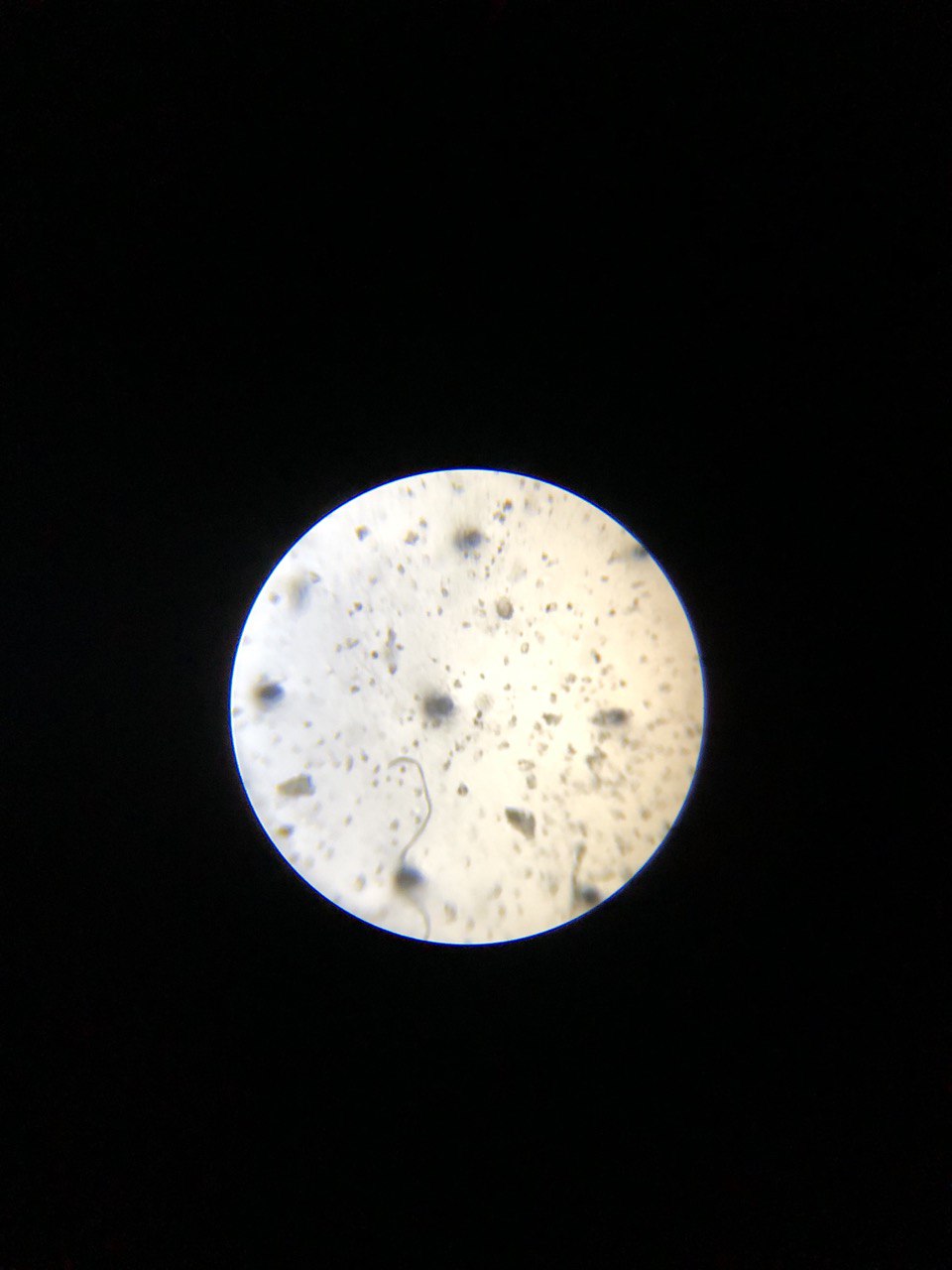

Supplement: Supplementary file 9 — Supplementary Data 1 [file 44172_2024_294_MOESM9_ESM.zip › Sourcce Data/Raw Microscopy images/holes made by MNs using spring applicator.jpg]

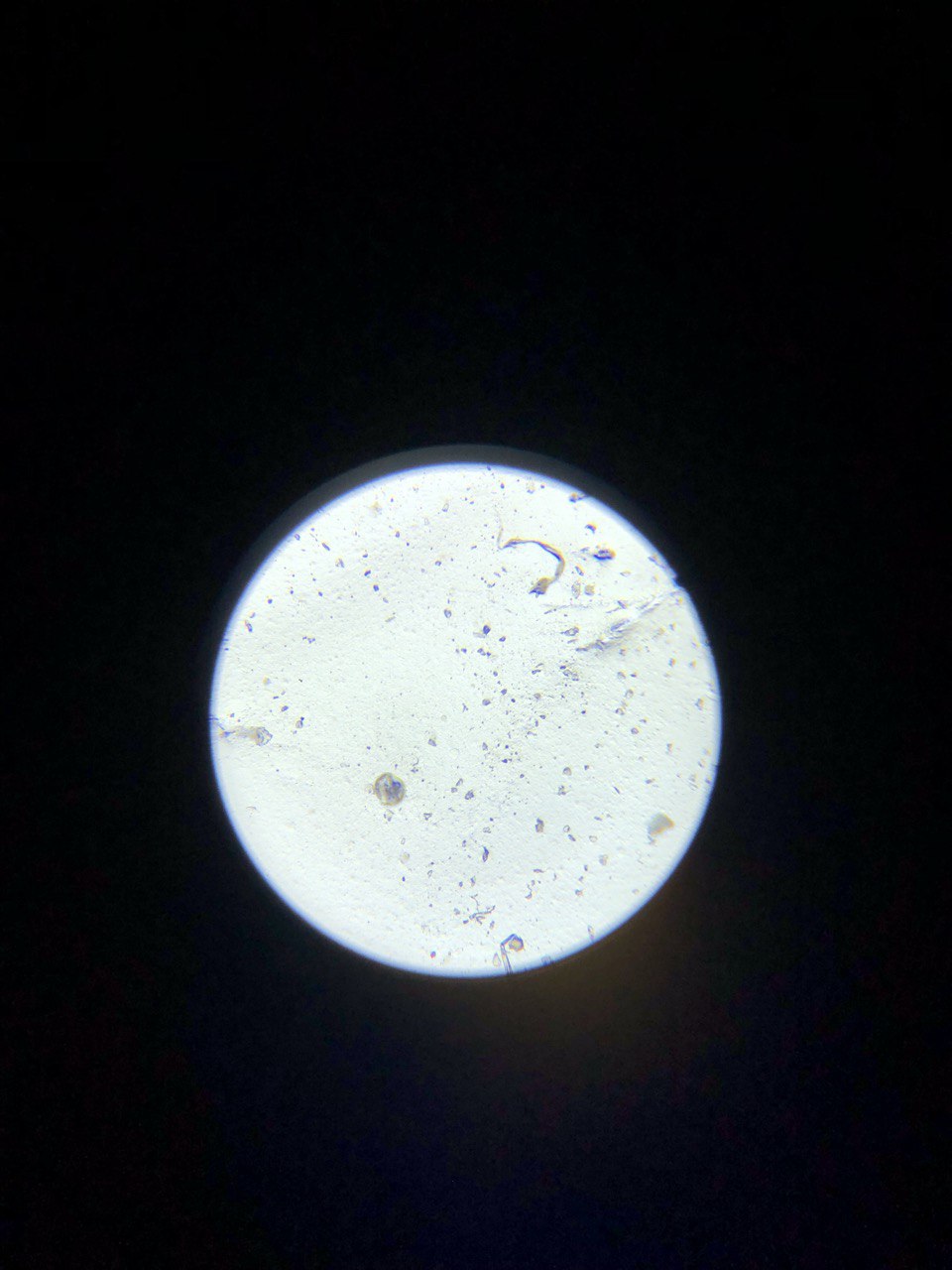

Supplement: Supplementary file 9 — Supplementary Data 1 [file 44172_2024_294_MOESM9_ESM.zip › Sourcce Data/Raw Microscopy images/holes made by origami1.jpg]

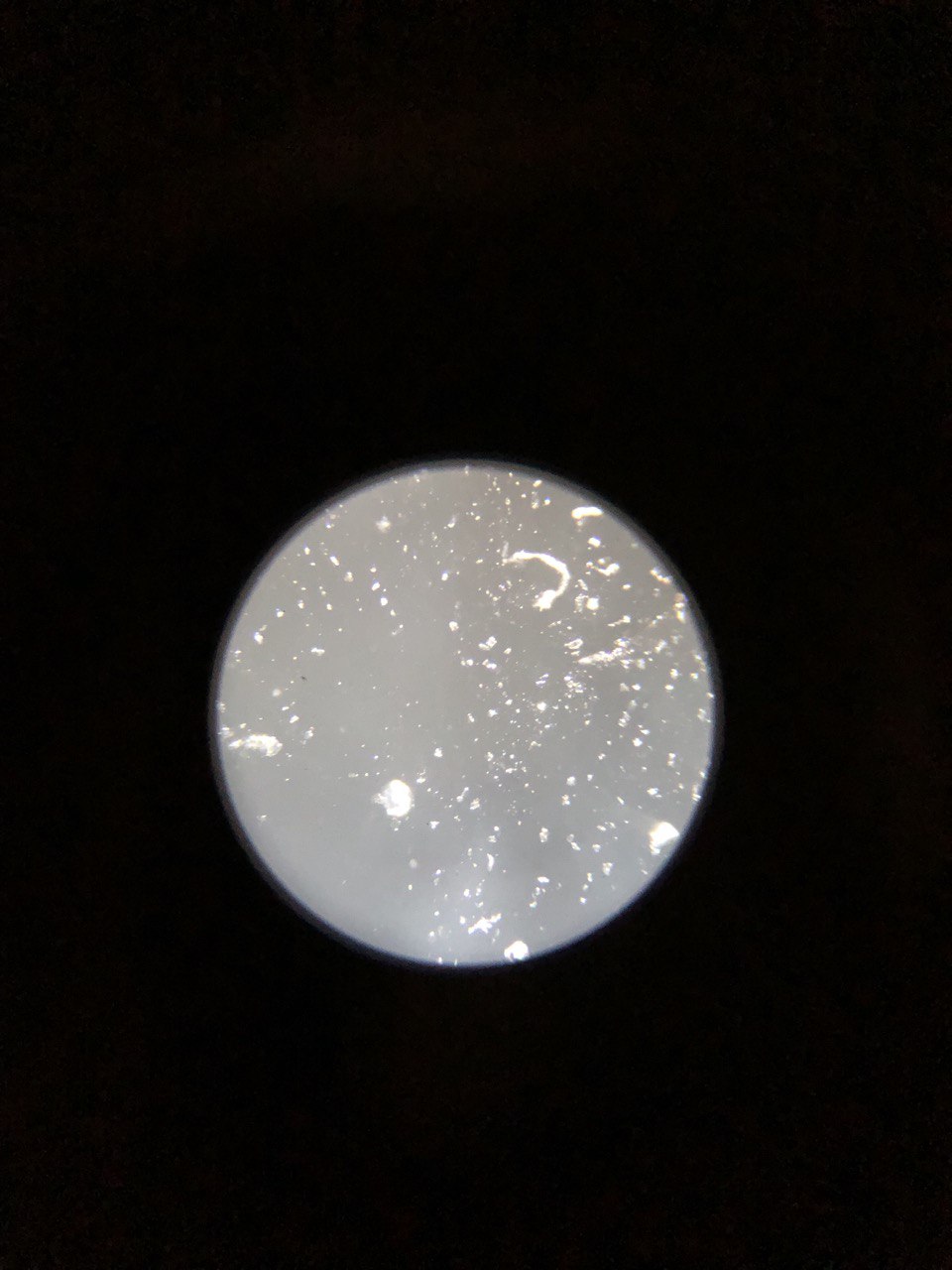

Supplement: Supplementary file 9 — Supplementary Data 1 [file 44172_2024_294_MOESM9_ESM.zip › Sourcce Data/Raw Microscopy images/holes made by origami2.jpg]

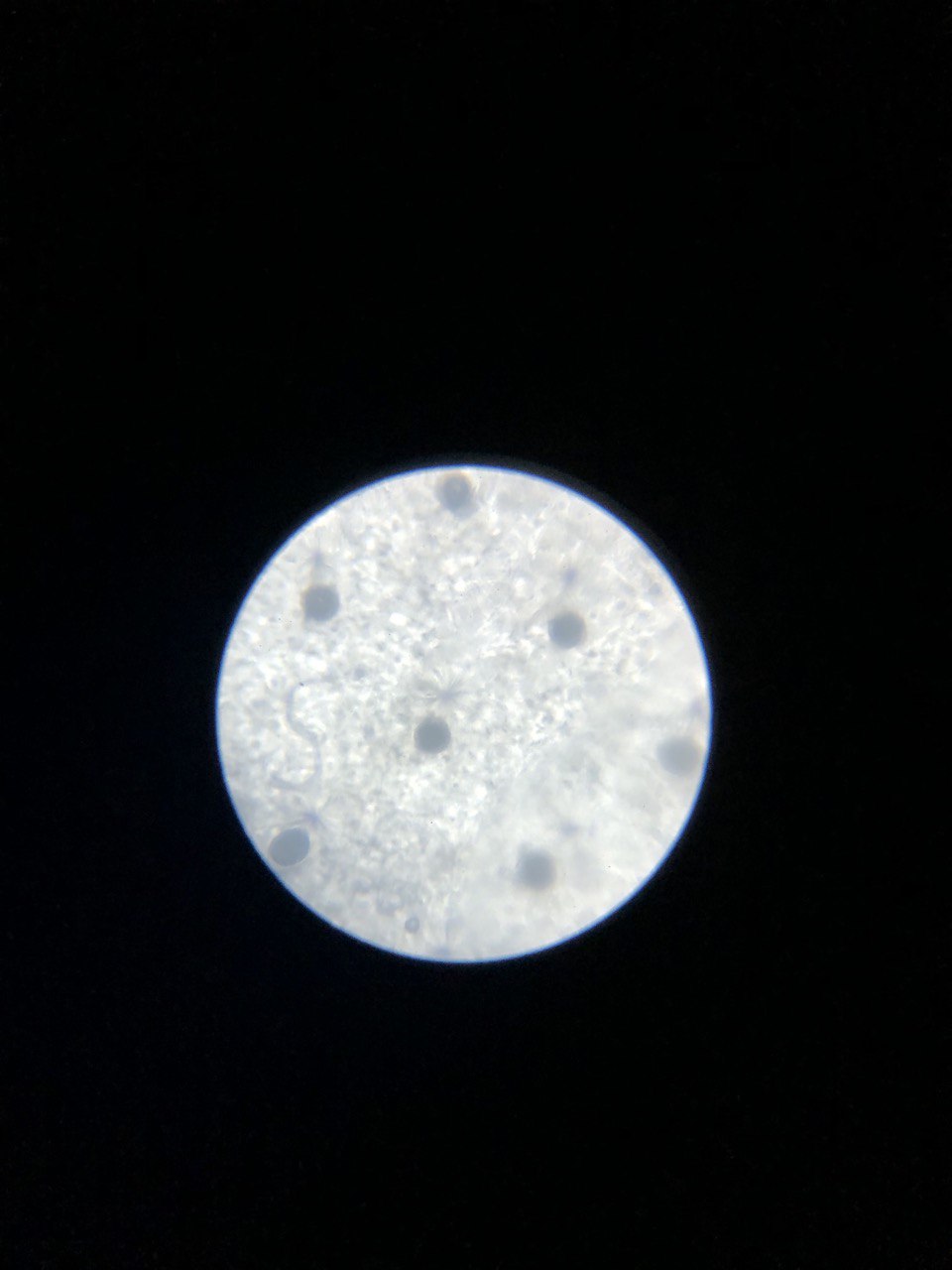

Supplement: Supplementary file 9 — Supplementary Data 1 [file 44172_2024_294_MOESM9_ESM.zip › Sourcce Data/Raw Microscopy images/Microneedles topology.jpg]

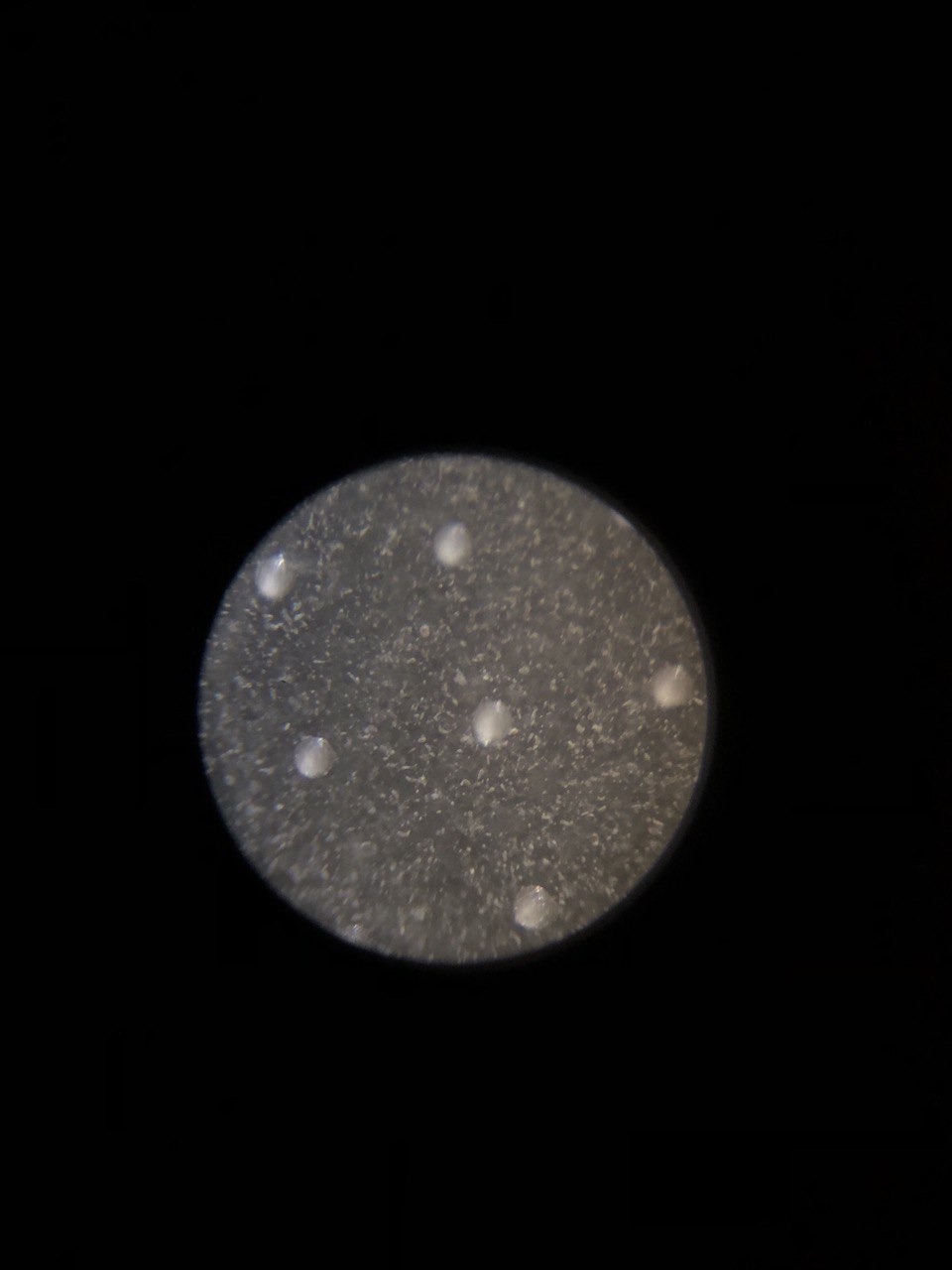

Supplement: Supplementary file 9 — Supplementary Data 1 [file 44172_2024_294_MOESM9_ESM.zip › Sourcce Data/Raw Microscopy images/MN topology 2.jpg]

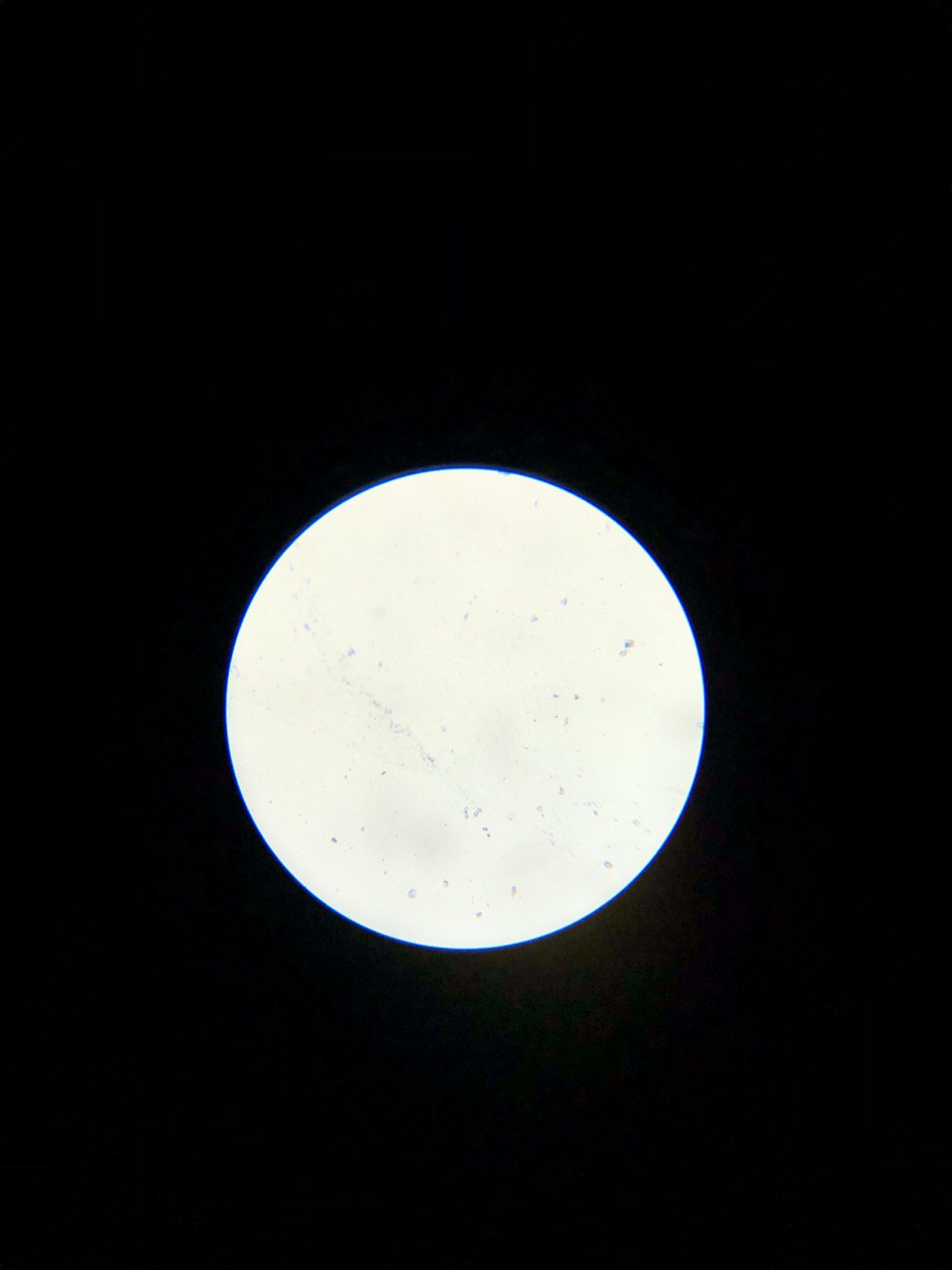

Supplement: Supplementary file 9 — Supplementary Data 1 [file 44172_2024_294_MOESM9_ESM.zip › Sourcce Data/Raw Microscopy images/Plain ecoflex.jpg]
